# Intelligent automation cognitive system for accurate malaria diagnosis using digital blood smears

**DOI:** 10.1371/journal.pone.0348280

**Published:** 2026-06-18

**Authors:** Emad Malaekah, Husham Saied, Othman Alfahad, Tatyana Utkina, Marwa A. Saleh, Ahmad abduaziz Aljaffer

**Affiliations:** 1 Department of Biomedical Technology, Prince Sultan Military College of Health Sciences, Dammam, Saudi Arabia; 2 Department of Robotics and Specialized Computer Systems, Cherkassy State Technological University, Boulevard, Cherkassy, Ukraine; 3 Faculty of Pharmacy (Girls), Pharmaceutical Organic Chemistry, Al-Azhar University, Cairo, Egypt; 4 Department of Clinical Laboratory, Prince Sultan Military College of Health Sciences, Dammam, Saudi Arabia; Para Federal University, BRAZIL

## Abstract

Malaria is a potentially fatal illness caused by a parasite of the genus Plasmodium that humans get by being bitten by female Anopheles mosquitoes carrying the infection. The incidence of malaria worldwide is disproportionately high in the African continent. Automated systems and cognitive analysis of digitized images of blood smears were used to diagnose Plasmodium malaria. This method is implemented in the Aidos intelligent system, which is easily accessible online. For the study, the database included images of 191 blood smears of patients infected with malaria and 227 images of blood samples from healthy patients. The images were digitized using the method developed by Professor Lutsenko E.V. The images were digitized for 12 light spectra. Then, spectral analysis of the blood smear images was carried out only for 18 new patients, and the duration was 10 seconds. The average similarity value of Plasmodium malaria recognition in patients was achieved at 66.965%. No false positive decisions were obtained for digitalized blood smears from healthy patients. The automated system-cognitive analysis of digitized blood smears provides instant diagnostic support. It allows medical workers with limited knowledge in microscopy and artificial intelligence to perform diagnostics.

## 1. Introduction

Humans contract malaria via the bites of female Anopheles mosquitoes carrying the tiny parasite called *Plasmodium* that causes the sickness. A person’s red blood cells are destroyed by the *Plasmodium* malaria parasite, which then replicates in other cells. Typical symptoms of malaria include fever, headache, muscle soreness, and exhaustion [1]. WHO estimates that there will be 282 million cases of malaria and 610,000 deaths in 2024 – about 9 million more than the previous year [2].

A correct diagnosis at an early stage will reduce the cost of malaria treatment. *Plasmodium* malaria is detected by examining a drop of a patient’s blood, spread as a “blood smear” on a microscope slide, under a microscope [3]. Modern imaging techniques make digitizing microscopic blood smears with high optical resolution possible. A decision support system that assists microscopists in malaria diagnosis is available using computer vision to screen digital thin blood films, but it is prone to false negative decisions [4]. To count the number of infected cells in a single blood thick film, a skilled microscopist usually takes 20–30 minutes to carefully examine the film. *Plasmodium* malaria screening becomes a lengthy and error-prone process when there are many patients, and manual blood film evaluation mostly depends on skilled staff. When evaluating treatment results and the effectiveness of antimalarial medications, the parasite clearance rate is crucial [5]. Rapid tests, which use a small kit to identify antigens from malaria parasites, can be used to identify malaria. Through a hole in the kit, a drop of blood is added, and the tests are conducted inside the apparatus, yielding findings quickly [6]. However, the rapid diagnostic tests (RDTs) is imprecise, and any incorrect result may impact patient treatment [7].

Automated methods can help many patients detect *Plasmodium* malaria quickly, cost-effectively, and accurately. Sophisticated image processing techniques using shape, color, intensity, size, and texture automate *Plasmodium* malaria detection [8,9]. In laboratories, peripheral blood smears are stained with Giemsa to improve the reliability of malaria diagnostics [10]. Giemsa staining allows one to detect morphological features present in certain types of samples and absent in others. Lasers with an energy of 80–95 nJ remove the blood plasma covering the infected blood cells of interest from blood smears [11]. Diagnostics for *Plasmodium* malaria should be accurate, identify parasites in areas where they are endemic, and produce fewer false negative results [12]. Oxford University’s Visual Geometry Group (VGG) created a convolutional neural network for large-scale image recognition [13]. There are systems for processing pictures of blood smears captured by a smartphone camera mounted on a microscope’s ocular [14,15]. Convolutional neural network (CNN)-based deep learning techniques for smartphones may identify malaria parasites in thick blood smears by extracting and categorizing features [16]. Images of disease-infected and uninfected blood cells are processed by CNN automatically, without the need for human assistance [17,18]. However, these methods and tools are not available for widespread use. Deep belief networks (DBNs), probabilistic generative models consisting of different layers of hidden variables with associations between layers, are used for malaria detection [19,20]. However, DBNs require a labor-intensive setup procedure to be applied. An imperative capsule neural network is used to detect malaria. However, processing data using this network takes more than 33 minutes [21]. The primary challenge in all published research utilizing artificial intelligence and digital microscopy is the absence of high-quality blood smears or thick films that can identify sparse low parasitemia [22,23].

For molecular diagnostics of malaria at the point of care, integrated point-of-care devices (Point-of-Care) are used. These are compact automated systems that detect DNA or RNA of Plasmodium parasites directly at the patient’s site (in a clinic, field hospital, or laboratory). They utilize molecular biology methods such as Polymerase Chain Reaction or Loop-Mediated Isothermal Amplification [24]. Although the devices are automated, humans still play a vital role in malaria diagnostics. A specialist collects a drop of blood from a finger or vein, places the blood in a cartridge, inserts the cartridge into the device, checks the expiration date of the reagents, and initiates the analysis. The physician must maintain sterile technique and consider the epidemiological situation, the patient’s symptoms, and the results of other tests.

Smoothing filters such as Gaussian, median, and geometric mean filters suppress noise in microscopic images [25]. Even though the use of computer diagnoses is expanding rapidly, there are still issues with identifying low malaria parasites in blood smear microscopic pictures [26].

For more than 30 years, Aidos, an open intellectual system, has been used with success. It can be applied to any subject where a scientist or practitioner uses the most recent advancements in artificial intelligence to solve difficulties in their line of work and continuously expand their expertise [27]. It is suggested that an automated system-cognitive (ASC) analysis, which is incorporated into the universal intellectual system Aidos and was created by Professor Lutsenko, be used to identify blood smears infected with malaria [28]. The Aidos system’s ASC analysis model is based on systemic fuzzy interval mathematics, which makes it possible to handle big, complicated datasets that are interconnected, noisy, and fragmented. It accepts data in a variety of units of measurement and measurement scales, including numeric, ordinal, and nominal [29]. The Aidos system is also used for intelligent image processing, i.e., for their digitization, creation of models of specific images, abstraction, identification, classification of generalized images and solving several other problems. as a result of generalization, the value of image features for their differentiation is revealed, as well as the degree of specificity of certain features for specific images [30]. This allows, without compromising the adequacy of the model, to remove low-value features from it, i.e., to carry out abstraction of generalized images, which subsequently ensures a reduction in the costs of various types of resources for collecting and processing graphic information.

The present work aims to develop an intelligent system that can provide effective preliminary diagnostics of malaria for use in areas with insufficient medical care.

## 2. Methods

### 2.1. Formalization of the subject area

**Fig 1:**
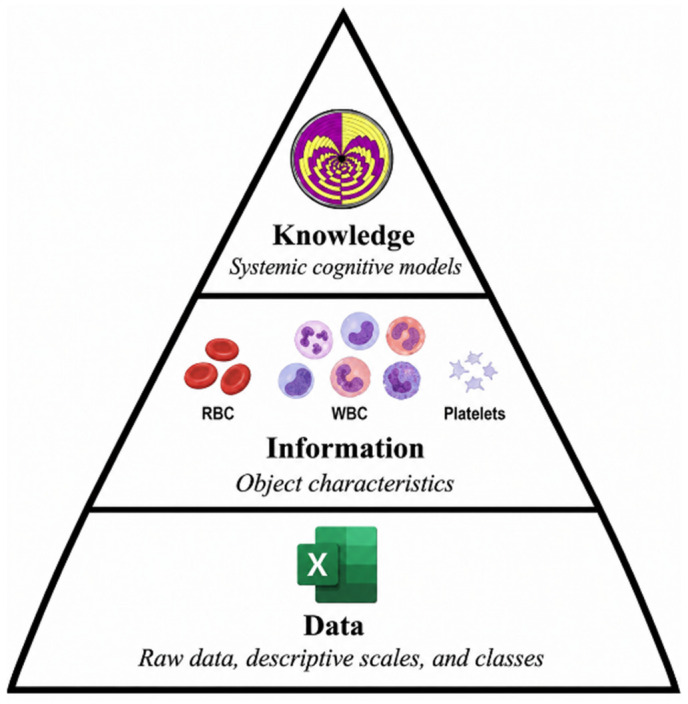
The process of transforming raw data into information and knowledge.

To study complex systems, it is necessary to transform source data into information, and then into knowledge. This knowledge is used to solve problems of classification, decision support, and domain research by exploring its system-cognitive model, generating a large number of tabular and graphical output forms. The process of transforming source data into information, and then into knowledge, is shown in Fig 1. For this study, it is necessary to first convert the graphic data into a text file.

**Fig 2:**
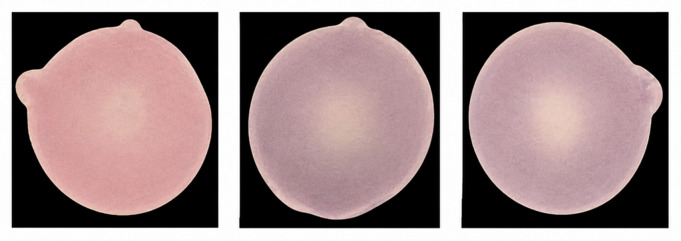
Malaria Plasmodium-negative blood smear samples.

**Fig 3:**
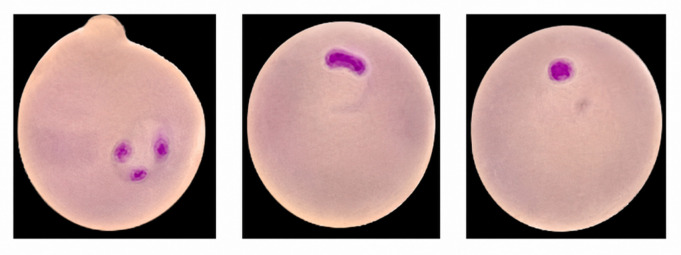
Malaria Plasmodium-positive blood smear samples.

Images for malaria diagnosis obtained by Machine Learning Engineer Nagadia from AURO University (Surat, Gujarat, India) and published online [31] were used for malaria diagnostics. This dataset contains 550 images of malaria (*Plasmodium*)-positive and negative blood smears, arranged in two folders, Train and Test, which can be used for training and testing, respectively. In this study, images from the Train folder were used. Examples of malaria-negative blood smear images are shown in Fig 2, and examples of malaria-positive are shown in Fig 3.

**Fig 4:**
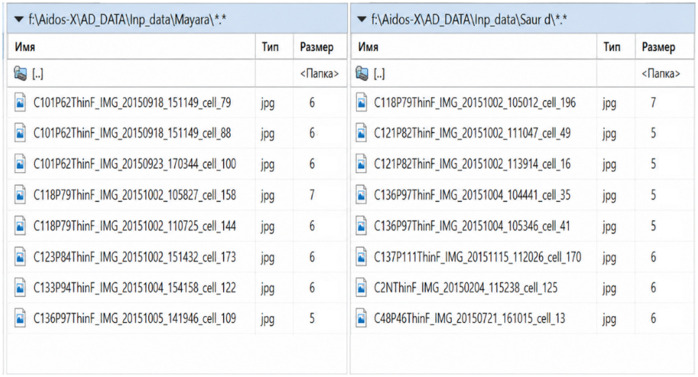
Structure of source image files.

To solve the problems in the article, we will use the software interface from the Aidos system to study images by their spectrum. To do this, download the Aidos system from the author’s website at the link: http://lc.kubagro.ru/aidos/_Aidos-X.htm and install it per the instructions on the website. Write the scanned images of blood smears to the folder  ..\AID_DATA\Inp_data\. A fragment of the structure of the original image files in JPG format is shown in Fig 4. The blood smears were not pre-processed and were used as they are posted in the Kaggle repository (https://www.kaggle.com/datasets/imdevskp/malaria-dataset). The blood smear photographs in the Kaggle repository dataset are publicly available, and the patient names are not provided, so no ethical approval or permission to use the samples for research is required.

**Fig 5:**
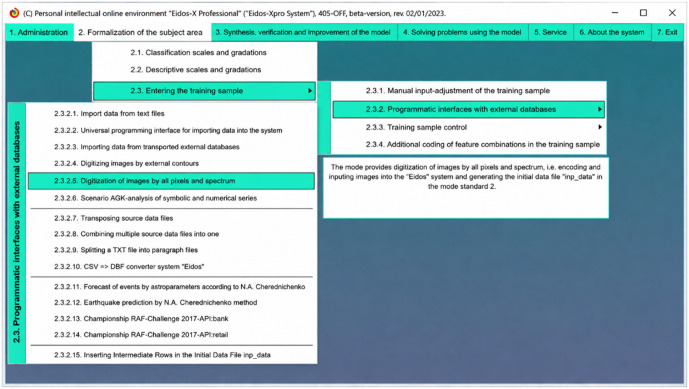
Interface of image processing mode by pixels and spectrum.

Mode 2.3.2.5 of the Aidos intelligent system was used to digitize the images. The appearance of this image processing mode is shown in Fig 5. This mode provides the conversion of images in BMP or JPG format into the lnp_data.xlsx source data file, in which a line represents each image. The subject area is formalized, models are created, and tested using this source data file.

The image digitization method developed by Professor E.V. Lutsenko [32] was used in this study. A patent protects the essence of the image digitization method by the intelligent Aidos system [33]. The method of digitizing images into a numerical feature vector proposed by Professor Lutsenko has the following features: the object’s contour is used; the image is converted to a polar coordinate system; a radial shape function is constructed; the obtained parameters are used as descriptors for image comparison. A typical algorithm (script) of the method consists of the following steps: scanning or photographing an object; converting the image to a standard format; performing object segmentation; obtaining a contour image. To convert the image to a standard format, the following are performed: centering the object, scaling it, and rotating it to a standard position. This makes the analysis independent of image orientation. The image center is taken as the origin of coordinates. For each pixel, its radial function is calculated, which determines: the average radius, dispersion, spectral characteristics, shape harmonics, and derivatives of the radial function. The resulting feature vector is used for classification, clustering, object identification, and constructing shape prototypes.

The Aidos system detects the colors in a picture with high accuracy by measuring the spectra of graphic objects. The picture spectrum is defined by the system as the percentage of pixels of various hues in the total number of pixels in the image. To measure the image spectra, the number of color ranges is specified. The Aidos system calculates the brightness of the Red, Green, and Blue rays to create a spectrum, and each color range represents a mix of these rays. The spectra of particular items are then compared with those of training classes. In this instance, the entire amount of information about an object’s color spectrum that pertains to its inclusion in the class’s generalized picture is computed. Digitizing 418 blood smear images was labor-intensive and took 1 hour 25 minutes on a computer with an AMD Athlon 3.1 GHz 32-bit processor and Windows 10.

Table 1 shows a fragment of the file  .. \AID_DATA\Inp_data\inp_data.xlsx with the results of digitizing images for twelve spectra. Column A shows the number of the objects of study, i.e., blood smears. Column B shows the codes of the analyses that correspond to the names of the files with the images of blood smears. When conducting a study in the Aidos system, the data in this column is not used, but it can be used for visual control of the correctness of the identification of objects.

**Table 1:** Fragment of a file with the results of digitizing images of blood smears.

Object	Analysis code	Class	SPECTRINTERV: 1/12	SPECTRINTERV: 2/12
A	B	C	D	E
**No1**	C133P94ThinF_IMG_20151004_155721_cell_111	Parasite	0.0000	0.0000
**No2**	C133P94ThinF_IMG_20151004_155721_cell_112	Parasite	0.0000	0.0825
**No3**	C133P94ThinF_IMG_20151004_155721_cell_113	Parasite	0.0000	0.0000
**No4**	C133P94ThinF_IMG_20151004_155721_cell_114	Parasite	0.0000	0.0000
**No5**	C133P94ThinF_IMG_20151004_155721_cell_115	Parasite	0.0000	0.6708

Column C is a classification and contains information about whether the patient is infected with malaria or not. The columns starting with column D are descriptive and contain digitized characteristics of the spectra of blood smear images.

### 2.2.  Synthesis of statistical and system-cognitive models

A formalized cognitive concept created by Professor E. V. Lutsenko enables the creation of mathematical models of the field under study. Conducting experiments reveals connections between elements: some elements are observed frequently, while others are rarely found together. Stable connections between elements suggest that they reflect an unavoidable reality, integral to these elements [34].

Mathematical models of the Aidos system are based on a matrix of absolute frequencies, reflecting the total counts, indicating the frequency of occurrences where levels of descriptive scales intersect with levels of classification scales (observations). But instead of using this matrix directly to solve every problem, matrices of conditional and unconditional percentage distributions are used. Calculated systemic cognitive models represent the data from experiencing a certain descriptive scale gradation, causing the modeling object to enter a state that corresponds to a specific classification scale (class) gradation. The models that are employed are A. Kharkevich’s measure of the amount of information, Karl Pearson’s χ-square, and the return on investment (ROI) coefficient, which is used in economics to manage an investment portfolio. The main idea behind these approaches is that the modeling object will be put into a particular state that corresponds to the class based on the quantity of information in the factor value. This makes it possible to process heterogeneous information about the states of the modeling object comparably and accurately [35].

Directly based on the empirical data, the matrix of absolute frequencies is calculated and presented in Table 2. Based on this, the matrix of conditional and unconditional percentage distributions is calculated and presented in Table 3. Then, based on Table 3, the matrices of systemic-cognitive models are calculated and presented in Table 4.

**Table 2:** Matrix of absolute frequencies (ABS statistical model).

		Classes					Sum
		*1*	...	*j*	*...*	*W*	
**Factor values**	***1***	$N_{11}$		$N_{1j}$w		$N_{1W}$	
	***...***						
	***i***	$N_{i1}$		$N_{ij}$		$N_{iW}$	$N_{i\Sigma }=\sum_{j=1}^{W}N_{ij}\text{\textbackslash{}hspace\{0.33em}\}$
	***...***						
	***M***	$N_{M1}$		$N_{Mj}$		$N_{MW}$	
**Total quantity** **signs by class**				$N_{\Sigma j}=\sum_{i=1}^{M}N_{ij}\text{\textbackslash{}hspace\{0.33em}\}$			$N_{\Sigma \Sigma }=\sum_{i=1}^{W}\sum_{j=1}^{M}N_{ij}$
**Total quantity** **learning objects** **samples by class**				$N_{\Sigma j}$			$N_{\Sigma \Sigma }=\sum_{j=1}^{W}N_{\Sigma j}\text{\textbackslash{}hspace\{0.33em}\}$

**Table 3:** Matrix of conditional and unconditional percentage distributions (statistical models PRC1 and PRC2).

		Classes					Unconditional probability sign
		*1*	...	*j*	*...*	*W*	
**Factor values**	***1***	$P_{11}$		$P_{1j}$		$P_{1W}$	
	***...***						
	***i***	$P_{i1}$		$P_{ij}=\frac{N_{ij}}{N_{\Sigma j}}$		$P_{iW}$	$P_{i\Sigma }=\frac{N_{i\Sigma }}{N_{\Sigma \Sigma }}$
	***...***						
	***M***	$P_{\text{M}1}$		$P_{Mj}$		$P_{MW}$	
**Unconditional** **probability** **class**				$P_{\Sigma j}$			

**Table 4:** System cognitive model matrix.

		Classes					Significance factor a
		*1*	...	*j*	*...*	*W*	
**Factor values**	***1***	$I_{11}$		$I_{1j}$		$I_{1W}$	$\sigma_{1\Sigma }=\sqrt[{2}]{\frac{\text{1}}{W-1}{\sum_{j=1}^{W}\left(I_{1j}-{\overset{―}{I}}_{\text{1}}\right)}^{\text{2}}}$
	***...***						
	***i***	$I_{i1}$		$I_{ij}$		$I_{iW}$	$\sigma_{i\Sigma }=\sqrt[{2}]{\frac{\text{1}}{W-1}{\sum_{j=1}^{W}\left(I_{ij}-{\overset{―}{I}}_{i}\right)}^{\text{2}}}$
	***...***						
	***M***	$I_{\text{M}1}$		$I_{Mj}$		$I_{MW}$	$\sigma_{M\Sigma }=\sqrt[{2}]{\frac{\text{1}}{W-1}{\sum_{j=1}^{W}\left(I_{Mj}-{\overset{―}{I}}_{M}\right)}^{\text{2}}}$
**Degree** **reduction** **class**	$\sigma_{\Sigma 1}$			$\sigma_{\Sigma j}$		$\sigma_{\Sigma W}$	$H=\sqrt[{2}]{\frac{\text{1}}{\left(W\cdot M-1\right)}{\sum_{j=1}^{W}\sum_{i=1}^{M}\left(I_{ij}-\overset{―}{I}\right)}^{\text{2}}}$

Designations in tables:

*i* – value of the past parameter;

*j* – value of the future parameter;

*Nij* – number of meetings of the *j*-th value of the future parameter with the *i*-th value of the past parameter;

*M* – total number of values of all past parameters;

*W* – total number of values of all future parameters.

*Ni* – number of occurrences of the *i*-th value of the past parameter throughout the sample;

*Nj* – number of occurrences of the *j*-th value of the future parameter throughout the sample;

*N* – number of occurrences of the *j*-th value of the future parameter with the i-th value of the past parameter throughout the sample.

*Iij* – a particular knowledge criterion: the amount of knowledge in the fact of observing the *i*-th value of the past parameter that the object will go into the state corresponding to the *j*-th value of the future parameter;

*Pi* – unconditional relative frequency of meeting the *i*-th value of the past parameter in the training sample;

*Pij* – the conditional relative frequency of meeting the *i*-th value of the past parameter at the *j*-th value of the future parameter.

For the INF3 model, which is most often the most reliable, the following partial χ-square criterion is used – the difference between the actual and theoretically expected absolute frequencies:


$$\begin{array}{c} I_{ij}=N_{ij}-\frac{N_{i}N_{j}}{N}. \end{array}$$

**Fig 6:**
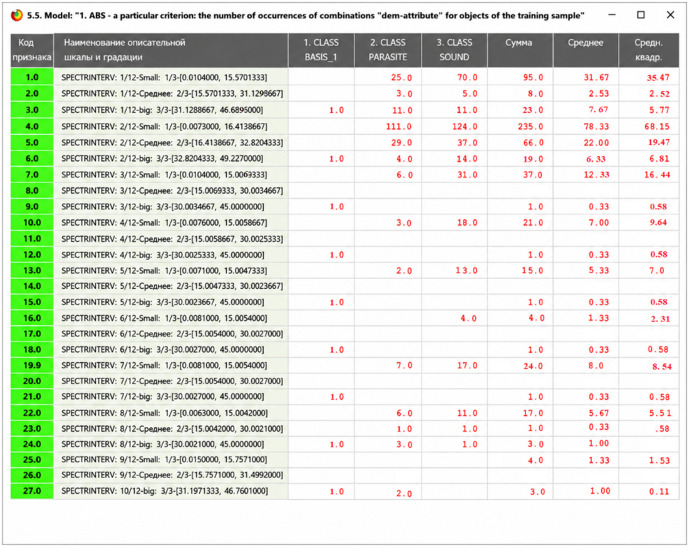
Matrix of absolute frequencies.

**Fig 7:**
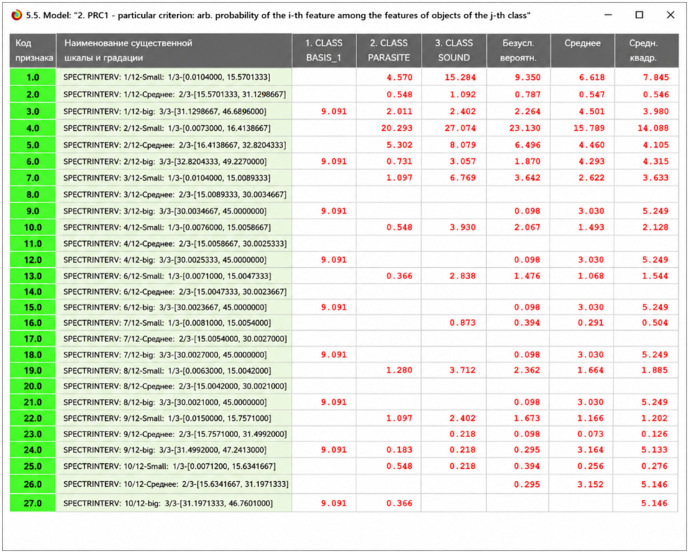
Matrix of conditional percentage distributions.

**Fig 8:**
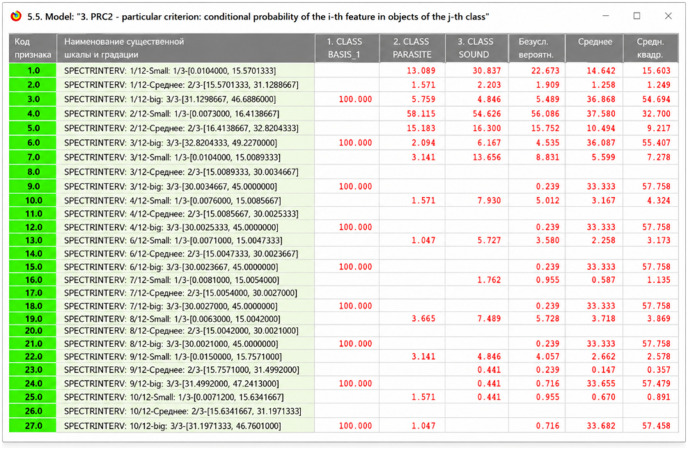
Matrix of unconditional percentage distributions.

Identification-related problems like classification, recognition, diagnosis, and prediction, as well as decision-making assistance and subject-domain exploration through examination of its system-cognitive representation, are successfully handled by using system-cognitive models [36]. Three statistical models and seven knowledge models can be used concurrently in the Aidos system, which aids in resolving issues with identification, decision-making, and subject-area study based on two essential criteria. The Aidos system assesses how well different integral and private criteria work to solve these issues. The absolute frequency matrix shown in Fig 6 was computed directly from the data collected during the digitalization of blood smear images. It served as the foundation for the calculation of the conditional and unconditional percentage distribution matrices, which are shown in Figs 7 and 8, respectively.

**Fig 9:**
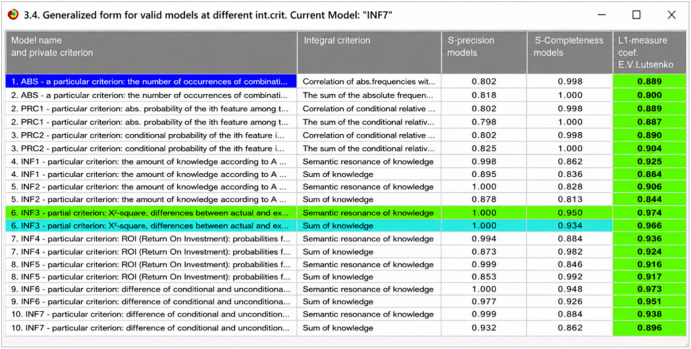
Generalized form of estimation of model reliability.

Typically, the acquired images have been contaminated by a variety of sounds, such as those caused by varying camera angles and microscope settings. To detect and reduce noise in the original data, as well as to identify disinformation in the original data and restore the real information, software tools are required. The training sample item is deemed atypical (an artifact) if the recognition results show that its degree of similarity with the class is below the desired threshold. There is a method for identifying, eliminating, and adding artifacts in the Aidos system. The percentage represents the threshold degree of similarity [37]. Fig 9 shows the model’s degrees of adequacy after removing artifacts with recognition accuracy below 25%. The L1 measure for the INF3 model, an information/knowledge model used to build “information portraits” and compute integrated similarity measures. Then equaled 0.976, which is a satisfactory result for medical intelligent applications.

The database includes digitized data of 191 blood samples of patients infected with malaria and 227 blood samples of healthy patients, and unrecognized digitized data were excluded from the database. To obtain an alternative solution, the virtual patient features equal to 45.0000 were added to the database containing digitized values of patient blood samples. If the database includes blood samples of only infected or only uninfected patients, the Aidos system will not be able to recognize them.

## 3. Results and discussion

### 3.1. Results of recognition of the examined blood smears

**Fig 10:**
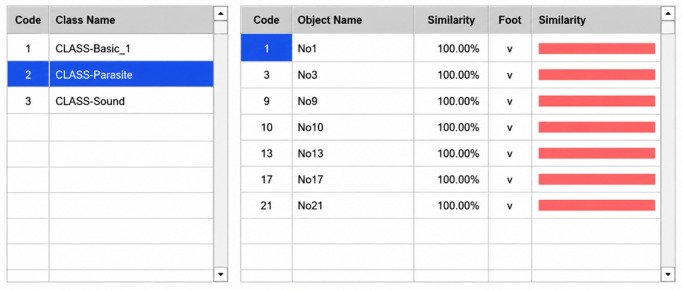
Visualization of malaria recognition results.

Using the INF3 model, digitalized blood smear image data were recognized with a high degree of similarity; examples of malaria recognition are shown in Fig 10. For several patients, a malaria recognition accuracy rate of 100% was achieved.

**Fig 11:**
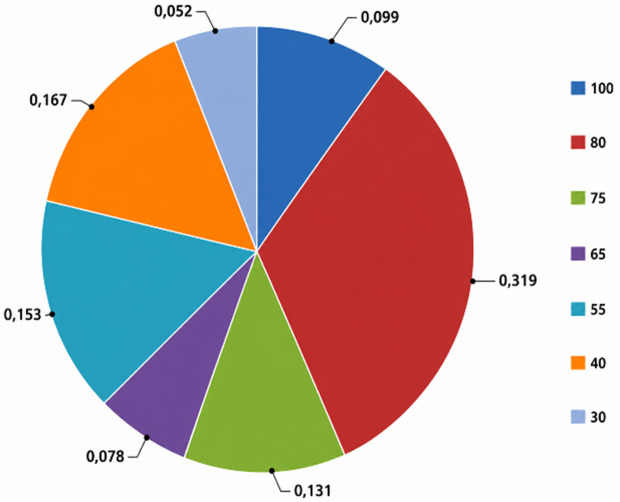
Diagrams of malaria blood sample recognition results.

The diagnostic accuracy for detecting malaria-infected blood smears was 100%. All infected samples were correctly recognized at varying similarity levels. Fig 11 presents a pie chart illustrating these recognition results: different colors represent different similarity ranges for malaria-positive samples. Notably, 31.9% of infected records were identified at a similarity threshold of 80%. Across the full database of malaria cases, diagnostic accuracy remained 100%, with an average similarity score of 66.97% compared with the reference “Parasite” class. In the right-hand column, color-coded bars display the achieved similarity levels for each detected malaria case.

**Fig 12:**
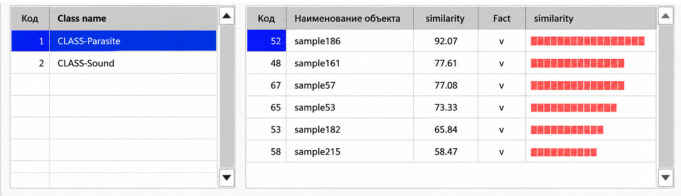
Results of recognition of digitalized blood smears of healthy patients.

Legend shows the levels of malaria detected in different colors. A fragment of the recognition of digitized blood smears of healthy patients is shown in Fig 12. The obtained similarity level is low. Still, the most important thing is that these samples are not classified as false positive solutions. The results of recognizing the absence of malaria in healthy patients are lower than those of known methods [17,18], for using the intelligent Aidos system, there is no need to purchase a license; it is available on the Internet and is recommended for use in developing countries.

### 3.2.  Results of the selection of light spectra for the study

**Fig 13:**
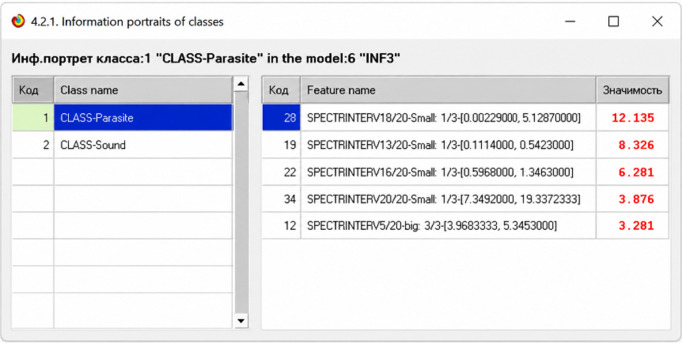
The importance of light spectra for detecting malaria.

The Aidos system allows for the determination of the light spectra that can detect malaria. The result of this spectrum is shown in Fig 13.

**Fig 14:**
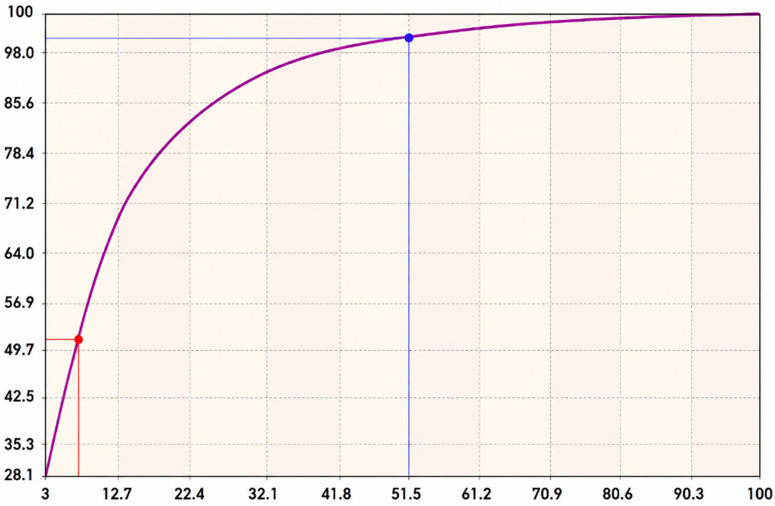
Pareto curve for the significance of descriptive scales. The Y-axis shows the total significance of the gradations of descriptive scales in %. The X-axis shows the gradations of descriptive scales in descending order of significance (as a percentage of their number).

Fig 14 shows the Pareto curve of the significance of descriptive scales (features) [38]. 7% of the most significant features provide 50% of the total importance. Half (50%) of the most considerable features provide 96% of the total importance. The number of gradations of descriptive scales can be significantly reduced without significant loss of model quality by removing insignificant gradations from the model. In this case, the dimensionality of the model will be significantly reduced, and its performance will increase accordingly.

### 3.3.  Results of SWOT-analysis

**Fig 15:**
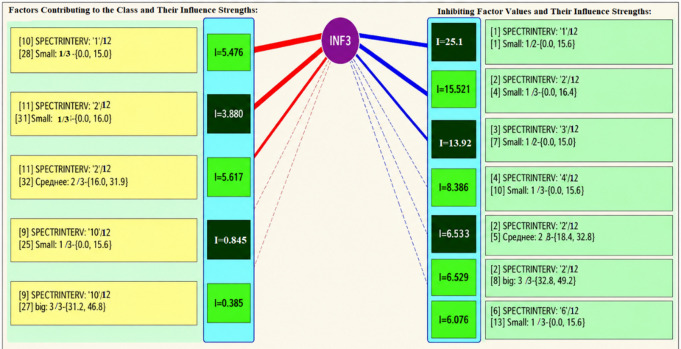
SWOT diagram of the Parasite class.

One well-known and accepted technique for strategic planning is SWOT (i.e., Strengths, Weaknesses, Opportunities, and Threats) analysis. This does not stop criticism, though, which is frequently valid, well-reasoned, and reasonable. The SWOT diagram for the Active Outcome class represents a feature contribution analysis specific to this class. In this context, SWOT is interpreted as an analytical framework highlighting the strengths, weaknesses, opportunities, and threats associated with feature–class relationships. The diagram illustrates the most significant associations derived from ASC analysis, where positive and negative contributions are encoded by color (red/blue), and the thickness of the connecting lines reflects the strength of each feature’s influence on the outcome. A thorough evaluation of SWOT analysis revealed numerous flaws, the main one being the requirement to consult specialists in order to determine the direction and magnitude of each factor’s influence [39]. Because of their expertise and professional experience, specialists do this instinctively. However, specialists’ talents are limited, and for a variety of reasons, they frequently are unable or unwilling to do this. Consequently, the challenge of performing a SWOT analysis without consulting professionals emerges [40,41]. By automating expert functions, such as determining the direction and magnitude of a factor’s influence based on empirical data, the Aidos system solves this issue [42]. The SWOT diagram of the Parasite class is shown in Fig 15.

The SWOT diagrams display the 12 most significant relationships, with the connection sign displayed in color (red plus, blue minus), and the value displayed by the thickness of the line. It is possible to display charts with only positive or only negative relationships. As a method for automated quantitative SWOT analysis, quantitative SWOT analysis of classes allows for the creation of a SWOT matrix for a particular class, indicating the degree of effect of facilitating and hindering elements directly based on the generated database. Factors that have a favorable impact on the control object’s transition to a certain state are listed in the class’s left column, followed by those that have no apparent impact and, finally, those that hinder the transition. It is feasible to identify the color spectrum range that will enable malaria to be reliably identified based on the findings of the SWOT analysis.

### 3.4. Limitations

Blood smear images must not exceed 800 x 600 pixels. Image digitization is performed using 12 spectral ranges. The Aidos system stores the intermediate data it can process in up to 2 GB of memory, limiting the number of images it can process. To ensure high reliability, blood smears should be of uniform size and shape (e.g., round) and use consistent staining and color hues.

Processing color images of blood smears is computationally intensive and demands substantial memory, as each fundus image contains hundreds of thousands of pixels. If large collections of smears are pre-digitized and stored, future spectral analysis needs to be applied only to new patient images, thereby shortening the malaria diagnostic process. To import converted data into the Aidos intelligent system, users must employ its universal software interface and specify the correct data format. All input files must be in Excel format. Although the current Aidos platform can handle millions of individual objects, its knowledge bases are limited to no more than 4,000 classes and 4,000 factor gradations.

Another significant limitation is the complexity of the Aidos user interface, which provides 55 operating modes (excluding the Exit mode). Even minor errors in data preparation or interface navigation can halt processing or generate inaccurate results. To reduce the learning curve, it is strongly recommended to review the training presentations available at [https://www.patreon.com/user?u=87599532], which explain the principles of automated system-cognitive analysis for solving complex diagnostic tasks [43].

## 4. Conclusion

A training database of blood smear parameters was successfully created. To evaluate its medical applicability, 18 test images were digitized—nine from malaria-infected patients and nine from healthy individuals. The digitization process for this test set of images took three minutes. The digitized data was saved to an Excel file. To digitize the test set of blood smear images, they were added in pairs to the training database, which required additional processing time. For large-scale malaria screening, new blood smear images should be added to the training database one by one, otherwise diagnostic reliability may be reduced. An expanded study of 436 digitized blood smears demonstrated 100% diagnostic accuracy, with all infected and healthy samples correctly classified. The number of blood smear images in the training set can be increased, but they must be digitized only once. Blood smear images in the test set can be digitized separately. This requires an additional step of merging the digitized data files from the training and test sets into a single Excel file. The additional one-time cost of digitizing a large number of training set images will only slightly increase the overall diagnostic time for test blood smear images.

Professor Lutsenko’s L1 measure of reliability of the INF3 model is 0.974. S-precision model INF3 equals 1.000, S-completeness equals 0.950. The confidence interval for malaria classification is defined by similarity values ranging from >25–100 when comparing digital smears with reference classes (parasites and healthy individuals). Images with a similarity score below 25 should be excluded from consideration as unrecognized. No such images were found in this study.

The Aidos intelligent system offers the following advantages over traditional microscopy:

- The system automatically detects red blood cells and parasites;
- Reduces the need for experienced microscopists;
- Eliminates laboratory technician bias;
- High diagnostic accuracy of blood smears, approximately 97–98%, is achieved, comparable to that of experienced parasitologists;
- Analysis is completed in minutes, whereas manual microscopy can take 20–30 minutes per sample;
- The system can automatically screen millions of cells, which is virtually impossible to do manually.

The Aidos intelligent system transforms malaria diagnosis from an expert manual procedure into an automated digital process. This is especially important for countries in Africa and Southeast Asia, which are remote from laboratories, and where experienced microscopists are often unavailable in endemic regions.

## Supporting information

S1 File Inp_data. (XLSX)
