# Supplementary material for: Intelligent automation cognitive system for accurate malaria diagnosis using digital blood smears
Source: PLoS One. 2026 Jun 18;21(6):e0348280. doi: 10.1371/journal.pone.0348280 (PMC13278421; doi:10.1371/journal.pone.0348280)
Supplement: Attachment — Submitted filename: Reviewers Comments.pdf [file pone.0348280.s002.pdf]

Dear Editor,

Please find below, my comments and suggestions on the reviewed manuscript:

General:

The manuscript describes a study which employed an automated system-cognitive analysis of digitized blood smears as a diagnostic tool for malaria. This is a step forward in malaria diagnosis. However, there is the need for a critical revision of the manuscript to attend to the reporting style in order to ensure the validity and reliability of the findings. There is also a need for clarity and consistency throughout the manuscript so as to strengthen its overall quality and contribution to the understanding of the use of automation cognitive system for digital blood smears in malaria diagnostics.

- The English language quality of the manuscript needs improvement to make for better reading.
- The technical language of the manuscript needs to be revised to make it more scientifically sound, and easily comprehensible across the readership community.
- The authors need to state/include at least, the genus of the causative agent(s) of malaria (i.e., ***Plasmodium***) in the manuscript. This can be used interchangeably with malaria where applicable.

Graphical Abstract:

- Line 1: The authors should consider including the highlighted words: "…. by **species of *Plasmodium*** parasites …"
- Line 2: The genus name, ***Anopheles***, should be italicized; and subsequently, throughout the manuscript.

Introduction:

- Line 1: "**contract**" is a better word for ''obtain''. Also consider including the highlighted texts: "…. female ***Anopheles*** mosquitoes carrying ….."
- Lines 3-5: The statement "According to the ……., 438,000 … in 2015, … 620,000 … in 2017, …. 300-500 million …… [2]" is obsolete and should be replaced with more recent data. The authors are advised to refer to: **(263 million and 597,000 malaria cases and deaths respectively, in 2023 [World Malaria Report, 2024]).**
- Line 12: The authors need to be more specific with the statement "To count the number of infected cells in a single blood film …." by indicating the type of cells and blood film (thin or thick)?
- Lines 16-19: The statement "Rapid tests …….. impact patient treatment [7]." should be recast for better comprehension. For instance, "Rapid tests" could be re-written as "**rapid diagnostic tests (RDTs)"**
- Third paragraph: The authors need to be more specific with the statement: "Sophisticated image processing ….. automate malaria detection [8]". ….. using shape, color, intensity, size and texture …..". The question is: Of what? The malaria parasite, or blood cells?
- Third paragraph: The authors need to be more specific with the statement: "Lasers …. the cells of interest from blood smears [8]". The authors should state the cells of interest.
- Third paragraph: Consider revising the statement as highlighted: "Images of **diseased (infected)** and uninfected **red** blood cells **(RBCs)** … [16, 17]".
- Third paragraph: Consider revising the statement as highlighted: " … films that can identify **low** parasitemia [21, 22]".
- Third paragraph. If what the authors mean by tiny in the phrase? … with identifying **tiny** malaria parasites in blood … [24]" is in regard to the malaria parasite density, then they should consider using **low**.
- Page 4: Line 2: "….. **and solving several other problems.**" should be referenced.
- Last paragraph. Consider the following "The **present** work aims to ….".

Methods

2.1. Formalization of the subject Area:

AURO University is in India. However, it is not in Mumbai, Maharashtra, as stated by the authors, but rather in **Surat, Gujarat**.

- Consider revising "…. 550 images of infected and uninfected blood smears ….'' as: "…. 550 images of **malaria (*Plasmodium*)-positive and negative blood smears**, ….''
- ….. can be used for training and testing. **Respectively**???
- "Examples of malaria-**unaffected** ….'' to "Examples of malaria-**negative** ….''
- "… examples of **affected** …. are **demonstrated** ….'' to "… examples of malaria-**positive** …. are **shown** ….''
- Consider revising Titles of Figs. 1 and 2 as **Malaria/*Plasmodium*-negative** blood smear samples and **Malaria/*Plasmodium*-positive** blood smear samples, respectively.
- Page 5, lines 7/8: "**The spectra ….. are then compared with those of classes**''. What classes? According to the Eidos system or what?
- Last paragraph: "…. starting with column **Dare** …'' to "…. starting with column **D are** …''

2.2. Synthesis of statistical and system-cognitive models:

- Consider revising Title of Fig. 6 as **Matrix of conditional percentage distributions**.
- Page 8: Line 9: What is the full meaning of the acronym **INF3**?
- Page 9: Line 5: Consider replacing '**sick**' with '**infected**' and '**healthy**' with '**uninfected**'.

Results and Discussion

3.1. Results of recognition of the examined blood smears:

- Fig. 9: Given that a malaria recognition accuracy rate of 100% was recorded with several of the studied smears, the authors should consider showing at least, one of such in Fig. 9.
- "**All 100%** of infected ……'' Better to use either of the two (i.e., **All** or **100%**).
- Consider changing "The **circular histogram** of malaria ….'' to "The **pie chart** of malaria ……''
- Fig. 10: This is not a **circular histogram**, but rather, a **pie chart**! Also, the **,** (commas) in the listed values should be replaced by **.** (i.e., dots).
- Page 9, Last paragraph: Consider replacing "**The right column ……. colors**'' with "**Legend:**''. This should be placed atop the column showing the different colors and values.
- Fig. 11: The data shown here is a duplication of **Fig. 9**! Moreover, Fig. 11 is supposed to show the results of recognition of digitized blood smears of healthy patients!

3.2. Results of the selection of light spectra for the study:

- Consider revising "The Eidos system allows **you to determine** …..'' as "The Eidos system allows **for the determination of** …'' and "The **effect** of light spectra is …..'' as "The **result of this** is …….''
- Page 11, Line 1: The full meaning of SWOT (i.e., **Strengths, weaknesses, Opportunities, and threats**) should be stated in parenthesis.

Conclusion

- Line 6: i.e., infected blood masks?
