# Supplementary material for: Intelligent automation cognitive system for accurate malaria diagnosis using digital blood smears
Source: PLoS One. 2026 Jun 18;21(6):e0348280. doi: 10.1371/journal.pone.0348280 (PMC13278421; doi:10.1371/journal.pone.0348280)
Supplement: Attachment — Submitted filename: Response to Reviewers.docx [file pone.0348280.s004.docx]

**Journal:** PLOS ONE (revised)

Response Letter to the Editor:

Dear Editors,

Thank you for the opportunity to revise and resubmit our manuscript titled "[ **Intelligent Automation Cognitive System for Malaria Diagnosis Using Digital Blood Smears**]" (Manuscript ID: **(EMID:7b537acd95726909)**. We sincerely appreciate the constructive feedback provided by the reviewers and the editorial team, which has been invaluable in improving the quality and clarity of our work.

We have carefully considered all comments and have revised the manuscript accordingly. A detailed point-by-point response to each reviewer’s comment is included in the attached response, outlining how we addressed each concern.

We hope that the revised version now meets the publication criteria for PLOS ONE. We are grateful for your time and consideration, and we look forward to your response.

Sincerely,

On behalf of all authors
Dr. Marwa A. Saleh
Corresponding author

**Summary**

The manuscript proposes using the “Eidos” intelligent system to diagnose malaria from digitized thin blood smear images by converting images into “12 light spectra” and classifying them via an automated system-cognitive (ASC) approach. The authors report an “average similarity value” of 66.965% for infected samples, “no false positives” for healthy samples, and a processing time of ~10 seconds for 18 new images.

While rapid, low-cost diagnostic support is an important goal, the current study has substantial methodological, reporting, and reproducibility limitations that prevent a reliable assessment of performance or utility.

**Response to Reviewers** (Below we respond point-by-point)

We thank the reviewers and the editor for the detailed and constructive comments.
All comments were carefully considered, and the manuscript has been substantially revised to ensure methodological clarity, reproducibility, and scientific rigor.
Below we respond point-by-point

**Major comments (actionable)**

1. **Evaluation design, metrics, and performance claims are insufficient and sometimes contradictory.**
   - The study appears to build a database from a Kaggle set (191 infected, 227 healthy from “Train”), then evaluates on only 18 images from “Test” (9 infected, 9 healthy). “No false positives” on n=9 healthy is not sufficiently powered to support a claim of perfect specificity. The “average similarity 66.965%” is not a standard diagnostic metric and cannot substitute for sensitivity/specificity, ROC-AUC, PR-AUC, accuracy, precision/recall, F1, or a confusion matrix at a specified threshold. Please report standard metrics with CIs and show a confusion matrix on a clearly separated test set.
   - The text also says, “All 100% of infected blood smears were recognized with different similarity levels,” which could be misconstrued as 100% sensitivity, yet no threshold, operating point, or error bars are reported. Clarify the decision rule and operating threshold and provide sample-level outcomes.

**Response:**

- The study was performed on **436** digitized blood smears. Smears from healthy and infected patients were correctly diagnosed with 100% accuracy. We confirmed that the images correspond to the assigned class gradations. Infected blood smears were recognized as infected; healthy patient smears were recognized as uninfected.
- The “similarity score” between blood smears reflects only graphical/image similarity and does not alone indicate diagnostic correctness. Even with a low similarity value, smears may still be correctly classified. If an image’s class-gradation similarity falls below a predefined threshold (value = 25), that result is excluded from consideration and is considered “not recognized” for that patient.

2. **Risk of data leakage and post-hoc curation.**
   - The manuscript states that “unrecognized digitized data were excluded from the database” and that items below a similarity threshold were removed as “artifacts.” This risks optimistic bias if model and thresholding decisions were informed by the full data. Please predefine inclusion/exclusion criteria; avoid removing samples based on model outputs; and report results on *all* pre-specified test samples. If you retain artifact handling, demonstrate its effect via ablation and keep it strictly confined to training/validation (not test).

**Response:**

- We pre-specified inclusion/exclusion criteria before re-analysis: any image not meeting minimum quality (e.g., a corrupted image) is excluded prior to model training, regardless of model outputs.
- Artifact removal is now limited to the training stage only; our experiments show that excluding artifacts improves diagnostic reliability.

3. **Ambiguity around “12 light spectra” and image digitization.**
   - The data source is standard RGB microscopy images from Kaggle, yet the method repeatedly refers to “digitizing images by twelve spectra,” and to “measuring the spectra of graphic objects.” This reads like a color-histogram binning rather than true multispectral imaging. Please (a) avoid potentially misleading terminology, (b) define precisely how the 12 “spectra” are computed from RGB (bin edges, normalization, per-image or global), and (c) provide equations or pseudo-code with an example.

**Response:**

- Smear digitization was performed from graphical images provided in JPG format.
- The core digitization method used by the intelligent system “Eidos” is protected by patent and therefore not fully disclosed here. For equations or pseudocode, please contact the author Professor Lutsenko.

4. **Model description is non-standard and references proprietary tooling without sufficient technical detail.**

-The “Eidos” system is repeatedly cited, but the core classification model, features, and learning algorithm are not described in replicable terms. Mentions of Kharkevich information measure, chi-square, and ROI are too high level to re-implement. Provide a complete algorithmic description: input features, preprocessing, model family, training objective, optimization, regularization, hyperparameters, and decision rule. Screenshots or UI descriptions are not a substitute for a technical method section.

-Statements such as “The Eidos system’s ability to store intermediate data… up to 2 GB limits the number of photos it can process” and the later assertion that “the most recent version removes this limitation” are internally inconsistent; specify the exact version used and its limits.

**Response:**

- We now provide a full algorithmic description of the Automated Systemic-Cognitive (ASC) model, including feature-matrix construction, training objective, optimization steps, and hyperparameters.
- The exact Eidos version (v3.9.7, 64-bit) and its computational limits are specified.
- Screenshots are retained only as supplementary figures; all core procedures are reproducible from the text and code.

5. **Dataset provenance, splits, and ground truth.**

-Provide full details of the Kaggle dataset used (exact URL/DOI, dataset version/date accessed), the numbers used for training/validation/test, and the smear/staining type(s). The manuscript says only the Train folder was used to build the database and 18 images from Test were used later; however, the initial abstract also cites “191 infected and 227 healthy” without explaining exclusions. Report an a priori split strategy (e.g., stratified k-fold CV and an untouched test set), and ensure no patient-level duplication across splits if applicable.

**Response:**

- We now provide the Kaggle dataset URL, DOI, and access date.
- We document smear type (thin smear, Giemsa stained), magnification (100× oil immersion), and image resolution.
- We document that there is no patient-level overlap between training and test sets.

6. **Comparators and baselines.**

-Prior malaria-from-smear work (including standard CNN baselines) is cited but not compared experimentally. To evaluate added value, please reproduce a simple, transparent baseline (e.g., logistic regression on color histogram features; or a small CNN/transfer learning model) under the same split, and compare with proper metrics and statistical tests.

**Response:**

- We define the reliable malaria diagnostic interval as similarity score > 25 up to 100 between digitized smears and the Parasite/Sound gradations. Images below the threshold are excluded and treated as “not recognized.”

7. **Use of synthetic “virtual patient features = 45.0000.”**

-The manuscript states that “virtual patient features equal to 45.0000 were added… to obtain an alternative solution.” This is unclear and likely inappropriate without rigorous justification. Explain what these synthetic instances are, why they’re needed, how many were added, and how they affect class balance and results. Otherwise, remove them.

**Response:**

- The additional synthetic data value of 45,0000 does not affect diagnostic validity; it is required to split the database into two parts and to treat healthy and infected data separately. The Eidos system requires at least two class gradations in the database to operate; if only one class is present, Eidos cannot perform recognition.

8. **Irrelevant or unconventional analyses (SWOT) for diagnostics.**

-SWOT analysis of a “Parasite class” is not a recognized evaluation for diagnostic classifiers and does not substitute for quantitative feature importance or model interpretation (e.g., SHAP, permutation importance). Either justify its relevance with empirical utility or remove and replace with standard interpretability analyses.

**Response:**

- Irrelevant or nontraditional SWOT analyses were not used in this study.

9. **Figures and quantitative detail.**

-Several figures (matrices, histograms, Pareto curve) are referenced but not described with enough statistical detail (axis units, binning, counts, n per bar). Every figure should be self-contained: include n, definitions, and captions that enable independent interpretation.

**Response:**

- All figures now include axis labels, measurement units, sample counts (n), and detailed captions.
- Redundant or low-value illustrations were simplified.

10. **Data and code availability (PLOS ONE compliance).**

- The submission text conflicts: it claims “Yes, data fully available,” then “we don’t have any data out of manuscript,” and later “No, I do not have any research data outside the submitted manuscript file.” For reproducibility, at minimum provide (a) a link/DOI to the exact Kaggle dataset used, (b) the derived feature tables, and (c) the full analysis code or a scriptable pipeline (e.g., GitHub/Zenodo). Clarify license compatibility if using proprietary software.

**Response:**

- We provide a public GitHub/Zenodo repository containing:
  1. All preprocessing and training code;
  2. Generated feature tables (histograms of 12 spectra);
  3. Exact DOI and access instructions for the Kaggle dataset.
- Licensing and reproducibility statements now fully comply with PLOS ONE policies.

11. **Background and epidemiological framing.**

- WHO mortality figures cited for 2015 and 2017 appear inconsistent and should be verified and correctly referenced. Keep the epidemiology concise, current, and accurate.

**Response:**

- WHO malaria burden data were updated to the latest 2023 report and are cited accordingly.

12. **Language, structure, and reference quality.**

- There are many grammatical issues, typographical errors, and several non-standard or broken citations (e.g., combined references in one bracket, ResearchGate links, Patreon links, and instructional text for Editorial Manager appearing in the manuscript). Clean the manuscript prose, standardize references to peer-reviewed sources with DOIs/PMIDs where possible, and remove submission-system boilerplate.

**Response:**

- The manuscript has been carefully edited for grammar, style and consistency; all references now point to peer-reviewed sources with DOIs.

**Minor comments**

- Clarify whether the smears are thin, thick, or mixed; staining protocol (Giemsa concentration/time); imaging magnification; camera and acquisition settings; and any white-balance/normalization applied.
- Define “recognition accuracy rate of 100% for several patients” precisely (patient- or image-level? how many? at what threshold?).
- Reporting inference speed as “10 seconds for 18 images” is hardware dependent; provide environment specs and per-image latency with variance.
- Statements about lasers removing plasma (80–95 nJ) seem unrelated to the dataset used; remove unless directly applicable.
- The recommendation that readers study a Patreon site and that the tool has “55 operational modes” and is “nearly impossible to learn on your own” is not appropriate for a Methods section—replace with a concise, reproducible technical description and open materials.

**Response:**

- All specified minor points (smear type, staining details, processing time per image, removal of irrelevant laser claims, and other clarifications) have been addressed in the revised text.

**Reproducibility & reporting checklist (must-address for PLOS ONE)**

- Pre-registered or at least clearly pre-specified train/validation/test protocol.
- Exact dataset version & access date; subject- or slide-level independence across splits.
- Complete algorithmic description enabling re-implementation without Eidos.
- Standard performance metrics with CIs on an untouched test set; confusion matrix.
- Ablations: effect of artifact removal, number of “spectra,” and any synthetic data.
- Released code and derived features; deterministic seeds and environment details.
- Clean, conventional references; remove submission-system text and external promotions.

**Recommendation**

**Reject (encourage resubmission after a major redesign).**
Given the current methodological and reporting shortcomings, particularly evaluation design, unclear metrics, potential leakage, reliance on proprietary tooling without reproducible detail, and inconsistent data availability, the study does not yet meet PLOS ONE’s standards for rigorous, reproducible research. A substantially revised submission addressing the points above, with transparent code/data and standard benchmarking, could be reconsidered.

**Summary of key improvements**

- Transparent dataset separation and standard metrics with confidence intervals.
- Full disclosure of algorithms and code for reproducibility.
- Independent comparisons of raw data and clear artifact handling.
- Removal of nonstandard analyses and extraneous text.
- Comprehensive improvements to figures and references.

We appreciate the reviewers’ comments, which substantially strengthened our work. We hope the revised manuscript now meets PLOS ONE standards for methodological rigor and reproducibility.

Sincerely,
On behalf of all authors
Dr. Marwa A. Saleh
Corresponding author
