# Supplementary material for: Intelligent automation cognitive system for accurate malaria diagnosis using digital blood smears
Source: PLoS One. 2026 Jun 18;21(6):e0348280. doi: 10.1371/journal.pone.0348280 (PMC13278421; doi:10.1371/journal.pone.0348280)
Supplement: Attachment — Submitted filename: REVIEWER REPORT.docx [file pone.0348280.s005.docx]

**REVIEWER REPORT**

**Manuscript Title:** Intelligent Automation Cognitive System for Malaria Diagnosis Using Digital Blood Smears

**Suggested Improved Title:** Automated Cognitive System for Accurate Malaria Diagnosis Using Digitized Blood Smears

**General Assessment:**

The manuscript presents the development and evaluation of an intelligent automation cognitive (ASC) system integrated into the Eidos platform for diagnosing malaria from digitized blood smear images. Using a database of 418 digitized blood smear images, the system demonstrated high diagnostic accuracy, achieving 100% correct classification for both malaria-infected and healthy samples. The study emphasizes automated spectral analysis of images, system-cognitive modeling, and quantitative feature selection, aiming to support healthcare workers with limited microscopy expertise in resource-constrained settings.

**Strengths:**

1. **Relevant and timely topic:** Malaria diagnosis remains critical in many endemic regions, and automation can significantly aid rapid and accurate diagnostics.
2. **Innovative methodology:** The use of the Eidos ASC system for image digitization, spectral analysis, and cognitive modeling is novel.
3. **High reported diagnostic accuracy:** Achieving 100% correct classification across 436 blood smear samples is highly promising.
4. **Practical applicability:** The system is free, web-accessible, and usable in low-resource settings.
5. **Comprehensive workflow description:** The manuscript clearly details the processes of digitization, system-cognitive modeling, feature selection, and recognition evaluation.

**Major Concerns / Points for Improvement:**

1. **Validation and reproducibility:**

a) Although 100% diagnostic accuracy is reported, the initial testing of new patients used only 18 samples. This raises concerns about overfitting and generalizability.
b) The proprietary nature of the Eidos system and its patented digitization method limits full reproducibility. Providing additional open methodological details or supplementary scripts would strengthen reproducibility.
c) Statistical validation metrics (e.g., sensitivity, specificity, precision, recall, F1-score, confidence intervals) should be added to provide a robust performance evaluation.

2. **Comparison with existing methods:**
   While the manuscript references deep learning approaches, it lacks a direct quantitative comparison with existing malaria detection methods. Including such a comparison would demonstrate the relative advantages of the proposed system.
3. **Discussion of limitations and deployment:**
   The computational and memory requirements, as well as interface complexity, are acknowledged. However, more discussion on real-world constraints—including variability in smear quality, lighting, staining, and clinical workflow integration—is recommended.
4. **Ethical considerations:**
   a) Explicit mention of ethical approvals or data usage permissions for patient samples should be included, even if the dataset is public.
   b) Potential clinical implications and how the automated system would support healthcare decision-making should be discussed.

**Minor Concerns:**
a) Some sentences are long and dense; the text could be simplified to improve readability.
b) Consistency in terminology (e.g., “Plasmodium malaria” → “Plasmodium spp.”) is recommended.

**Recommendations:**

**Decision:** Major revision required

**Suggested improvements before publication:**

1. Provide statistical validation metrics (sensitivity, specificity, precision, recall, F1-score, confidence intervals).
2. Include a comparative analysis with existing AI-based malaria detection methods.
3. Clarify ethical approvals and discuss clinical integration.
4. Provide additional methodological details or supplementary material to support reproducibility despite the proprietary Eidos system.
5. Improve clarity and readability, especially in the Methods and Introduction sections.

**Overall Assessment:**
The manuscript addresses a highly relevant problem and introduces a novel cognitive automation system for malaria diagnosis with promising results. However, improvements in validation, comparative benchmarking, reproducibility, and readability are necessary before acceptance.
