# Supplementary material for: Intelligent automation cognitive system for accurate malaria diagnosis using digital blood smears
Source: PLoS One. 2026 Jun 18;21(6):e0348280. doi: 10.1371/journal.pone.0348280 (PMC13278421; doi:10.1371/journal.pone.0348280)
Supplement: Attachment — Submitted filename: Responses to Reviewers.docx [file pone.0348280.s006.docx]

# Response to Reviewers

**Manuscript Title:** Intelligent Automation Cognitive System for Malaria Diagnosis Using Digital Blood Smears
**Journal:** PLOS ONE

Dear Academic Editor and Reviewers,

We sincerely thank you for your careful evaluation of our manuscript and for the constructive comments. We appreciate the opportunity to revise our work. We have carefully considered all comments and revised the manuscript accordingly. All changes have been incorporated into the revised manuscript and highlighted in the tracked-changes version.

Below, we provide a point-by-point response.

## Response to the Academic Editor

**Fund statement**

The funding has been removed, as the King Salman Center for Disability Research does not currently support this research due to exceeding the contractual deadline for completing the publication process.

**Comment:**
Please carefully review the comments provided by Reviewer Olaiya Folorunsho and incorporate the necessary revisions into the manuscript, particularly those concerning validation and reproducibility, as well as the discussion of limitations and practical implementation.

**Response:**
Thank you for this guidance. We revised the manuscript to strengthen the presentation of validation, reproducibility, limitations, and practical implementation. Specifically:

1. We clarified the dataset source and data usage conditions, including the public availability of the malaria blood smear images used in the study.
2. We explicitly stated that the study used a publicly available, de-identified dataset and that no institutional review board approval or informed consent was required.
3. We expanded the description of the digitization and analysis workflow, including the role of the Aidos system, the use of 12 spectral ranges, and the practical steps required for importing and processing images.
4. We added a clearer discussion of reproducibility-related issues, including file format requirements, image size constraints, computational limitations, and the need for consistent smear quality and staining.
5. We expanded the discussion of practical implementation by explaining how the system can support preliminary malaria screening and assist healthcare workers, especially in settings with limited access to expert microscopists.
6. We revised the limitations section to better reflect technical and operational constraints.

These revisions were incorporated into the Methods, Discussion, Limitations, Ethics statement, Source Data, and Conclusion sections.

## Response to Reviewer 1

We thank Reviewer-1 for the positive assessment and for confirming that the previous comments have been addressed.

### General comment

The graphical abstract needs proper labelling so that readers can quickly understand the process, key components, or findings.
Comment have been addressed

• The introduction part contains too much technical detail, and dense information such as laser energy in nJ, types of filters without context. It is better to write in a clear form for better interation with the reader.
Comment have been addressed

• The WHO death statistics quoted is old, use a recent report.
Comment have been addressed

• There is inconsistent flow in the write up: the transition from classical methods to deep learning is abrupt. Then the Eidos system is introduced without first motivating the gap that it fills. Give a brief introduction of Eidos and ASC.
Comment have been addressed

• Figure 1 and 2 looks blurred, replace with clearer images
Comment have been addressed
Results and Discussion

• You mentioned that using the INF3 model, digitalized blood smear image data were recognized with a high degree of similarity.
Give a clear explanation of this model before reporting it in this section.
Comment have been addressed

• You mention using SWOT analysis, can you give the full meaning and a brief description of the analysis.
Comment have been addressed

• Figure 13 needs to be properly labelled at the X and Y axis.
**Response:**
This comment has been addressed. Figure 14 has been updated with clearly defined axis labels to improve interpretability. Specifically, the X-axis now represents the gradations of descriptive scales arranged in descending order of significance (expressed as a percentage of their total number), while the Y-axis represents the cumulative significance of these gradations (expressed as a percentage).

We are grateful for your careful review and positive evaluation of the revised manuscript.

## Response to Reviewer 4

We sincerely thank Reviewer-4 for the valuable comments, which helped us improve the manuscript.

### Comment 4a

**Comment:**
Explicit mention of ethical approvals or data usage permissions for patient samples should be included, even if the dataset is public.

**Response:**
Thank you for this important comment. We have now explicitly clarified the ethical and data-use status of the dataset in the revised manuscript.

Specifically, we added that:

- The blood smear photographs were obtained from a **publicly available Kaggle repository;**
- The dataset is **de-identified**, and no patient names or personally identifiable information are provided;
- Therefore, **ethical approval and informed consent were not required** for the present study;
- Access to the dataset does not require approval from an ethics committee or any other institutional body.

These clarifications have been added in the **methods** section and further summarized in the **ethics statement** and **third party material**.

**Added text in the manuscript:**
“The blood smear photographs in the Kaggle repository dataset are publicly available, and the patient names are not provided, so no ethical approval or permission to use the samples for research is required.”

We also added in the Ethics statement section:
“This was not a human population study; therefore, approval by the institutional review board and informed consent were not required.”

### Comment 4b

**Comment:**
Potential clinical implications and how the automated system would support healthcare decision-making should be discussed.

**Response:**
Thank you. We have expanded the manuscript to better explain the clinical relevance and practical role of the proposed system.

In the revised Discussion and Conclusion, we now clarify that the proposed automated system is intended as a **preliminary diagnostic support tool**, not as a replacement for clinical judgment. The system can support healthcare decision-making in several ways:

The Aidos intelligent system offers the following advantages over traditional microscopy:

- The system automatically detects red blood cells and parasites;
- Reduces the need for experienced microscopists;
- Eliminates laboratory technician bias;
- High diagnostic accuracy of blood smears, approximately 97–98%, is achieved, comparable to that of experienced parasitologists;
- Analysis is completed in minutes, whereas manual microscopy can take 20–30 minutes per sample;
- The system can automatically screen millions of cells, which is virtually impossible to do manually.

We also clarified that the system provides **instant diagnostic support** and may be especially valuable in **Africa and Southeast Asia**, where access to specialized laboratory expertise is often limited.

**Added/strengthened text in the manuscript includes statements such as:**

- “The automated system-cognitive analysis of digitized blood smears provides instant diagnostic support.”
- “It allows medical workers with limited knowledge in microscopy and artificial intelligence to perform diagnostics.”
- “The Aidos intelligent system transforms malaria diagnosis from an expert manual procedure into an automated digital process.”
- “This is especially important for countries in Africa and Southeast Asia, which are remote from laboratories, and where experienced microscopists are often unavailable in endemic regions.”

We also expanded the limitations section to make clear that this system should be viewed as a supportive tool and that practical implementation depends on image quality, correct digitization, and proper operation of the Aidos interface.

## Additional Revisions Made in the Manuscript

In addition to the reviewer-specific comments above, we made several improvements for clarity and completeness:

- Clarified the description of the **INF3 model**, including its role as an information/knowledge model used for recognition and similarity assessment;

The description of the INF3 model has been refined, emphasizing its role as an information/knowledge-based model for recognition and similarity assessment. According to E. V. Lutsenko, the L1 reliability measure for the INF3 model is 0.974. The model demonstrates high performance, with S-precision equal to 1.000 and S-completeness equal to 0.950. Furthermore, the confidence interval for malaria classification is defined by similarity values ranging from >25 to 100 when comparing digital blood smears with reference classes (parasite-infected and healthy samples). Images with similarity scores below 25 are considered unrecognized and excluded from analysis. Notably, no such cases were observed in this study.

- Clarified the meaning of **SWOT analysis** as “Strengths, Weaknesses, Opportunities, and Threats” and briefly explained its use in the study;

The meaning of SWOT analysis (Strengths, Weaknesses, Opportunities, and Threats) has been explicitly clarified and contextualized within the study. The SWOT diagram for the Active Outcome class represents a feature contribution analysis specific to this class. It illustrates the most significant feature–class relationships derived from ASC analysis, where positive and negative contributions are encoded by color (red/blue), and the thickness of the connecting lines reflects the strength of influence.

- Updated and refined the **limitations** section;

The limitations section has been expanded and clarified. Specifically, blood smear images must not exceed a resolution of 800 × 600 pixels. Image digitization is conducted across 12 spectral ranges. The Aidos system can process intermediate data up to 2 GB of memory, which limits the number of images processed simultaneously. Additionally, to ensure high reliability, blood smear samples should be standardized in size and shape (e.g., circular) and prepared using consistent staining protocols and color characteristics.

- Ensured that the **Data Availability**, **Ethics statement**, and **Source Data** sections clearly describe the public dataset and its conditions of use;

The Data Availability, Ethics Statement, and Source Data sections have been revised to clearly describe the public dataset used and the associated conditions of use.

- Improved the language and organization of the manuscript to increase readability.

The manuscript has undergone thorough language editing and structural reorganization to improve clarity, coherence, and overall readability.

## Closing Statement

We appreciate the reviewers’ and editor’s thoughtful comments, which have helped us improve the manuscript substantially. We hope that the revised version now meets the journal’s requirements and is suitable for publication in PLOS ONE.

Sincerely,
**On behalf of all authors**
Dr. A. Saleh
