# Supplementary material for: Intelligent automation cognitive system for accurate malaria diagnosis using digital blood smears
Source: PLoS One. 2026 Jun 18;21(6):e0348280. doi: 10.1371/journal.pone.0348280 (PMC13278421; doi:10.1371/journal.pone.0348280)
Supplement: Peer review history

# Peer review: Decision Letter 0

**DOI:** 10.1371/journal.pone.0348280.r001

**Authors:** Segun OYEDEJI

8 Sep 2025

PONE-D-25-34548

Intelligent Automation Cognitive System for Malaria Diagnosis Using Digital Blood Smears

PLOS ONE

Dear Dr. Saleh,

Thank you for submitting your manuscript to PLOS ONE. After careful consideration, we feel that the study addresses an important health topic, but does not fully meet PLOS ONE’s publication criteria as it currently stands. Therefore, we invite you to submit a revised version of the manuscript that addresses the points raised during the review process.

==============================

First, there are substantial grammatical issues majorly bordering on the use of non-professional terminologies. I will suggest that the authors engage a Professional (biologist, parasitologist, epidemiologist or clinician) with sufficient kowledge of the disease to assist with the write-up.

Second, it was observed that standard performance metrics such as sensitivity, specificity, accuracy, precision, recall, F1 score, and ROC/AUC score, were not used to evaluate the result of the Model. The manuscript would improve significantly with the inclusion of the above standard performance metrics.

Third, evaluating only 18 images for the possibility of using the spectral analysis method for medical purposes is grossly inadequate. The authors could consider significant increase in this number to provide sufficient evidence to validate the results. The authors should also provide the procedure for this in the Methods Section.

Please go through each of the Reviewer’s Comments to address each point raised.

==============================

Please submit your revised manuscript by Oct 23 2025 11:59PM. If you will need more time than this to complete your revisions, please reply to this message or contact the journal office at plosone@plos.org. When you're ready to submit your revision, log on to https://www.editorialmanager.com/pone/ and select the ’submissions Needing Revision' folder to locate your manuscript file.

Please include the following items when submitting your revised manuscript:

- A rebuttal letter that responds to each point raised by the academic editor and reviewer(s). You should upload this letter as a separate file labeled 'Response to Reviewers'.
- A marked-up copy of your manuscript that highlights changes made to the original version. You should upload this as a separate file labeled 'Revised Manuscript with Track Changes'.
- An unmarked version of your revised paper without tracked changes. You should upload this as a separate file labeled 'Manuscript'.

If you would like to make changes to your financial disclosure, please include your updated statement in your cover letter. Guidelines for resubmitting your figure files are available below the reviewer comments at the end of this letter.

If applicable, we recommend that you deposit your laboratory protocols in protocols.io to enhance the reproducibility of your results. Protocols.io assigns your protocol its own identifier (DOI) so that it can be cited independently in the future. For instructions see: https://journals.plos.org/plosone/s/submission-guidelines#loc-laboratory-protocols. Additionally, PLOS ONE offers an option for publishing peer-reviewed Lab Protocol articles, which describe protocols hosted on protocols.io. Read more information on sharing protocols at https://plos.org/protocols?utm_medium=editorial-email&utm_source=authorletters&utm_campaign=protocols.

We look forward to receiving your revised manuscript.

Kind regards,

Segun Isaac OYEDEJI, Ph.D

Academic Editor

PLOS ONE

Journal Requirements:

When submitting your revision, we need you to address these additional requirements.

1. Please ensure that your manuscript meets PLOS ONE’s style requirements, including those for file naming. The PLOS ONE style templates can be found at https://journals.plos.org/plosone/s/file?id=wjVg/PLOSOne_formatting_sample_main_body.pdf and https://journals.plos.org/plosone/s/file?id=ba62/PLOSOne_formatting_sample_title_authors_affiliations.pdf

2. Please note that PLOS One has specific guidelines on code sharing for submissions in which author-generated code underpins the findings in the manuscript. In these cases, we expect all author-generated code to be made available without restrictions upon publication of the work. Please review our guidelines at https://journals.plos.org/plosone/s/materials-and-software-sharing#loc-sharing-code and ensure that your code is shared in a way that follows best practice and facilitates reproducibility and reuse.

3. When completing the data availability statement of the submission form, you indicated that you will make your data available on acceptance. We strongly recommend all authors decide on a data sharing plan before acceptance, as the process can be lengthy and hold up publication timelines. Please note that, though access restrictions are acceptable now, your entire data will need to be made freely accessible if your manuscript is accepted for publication. This policy applies to all data except where public deposition would breach compliance with the protocol approved by your research ethics board. If you are unable to adhere to our open data policy, please kindly revise your statement to explain your reasoning and we will seek the editor’s input on an exemption. Please be assured that, once you have provided your new statement, the assessment of your exemption will not hold up the peer review process.

4. We note that the grant information you provided in the ‘Funding Information’ and ‘Financial Disclosure’ sections do not match.

When you resubmit, please ensure that you provide the correct grant numbers for the awards you received for your study in the ‘Funding Information’ section.

5. Thank you for stating the following financial disclosure:

King Salman Center for Disability Research     KSRG-2024-281.

Please state what role the funders took in the study. If the funders had no role, please state: "The funders had no role in study design, data collection and analysis, decision to publish, or preparation of the manuscript."

If this statement is not correct you must amend it as needed.

Please include this amended Role of Funder statement in your cover letter; we will change the online submission form on your behalf.

6. Please note that funding information should not appear in any section or other areas of your manuscript. We will only publish funding information present in the Funding Statement section of the online submission form. Please remove any funding-related text from the manuscript.

7. Please amend your authorship list in your manuscript file to include author Marwa Saleh.

8. Please amend the manuscript submission data (via Edit Submission) to include author Marwa A. Saleh.

9. If the reviewer comments include a recommendation to cite specific previously published works, please review and evaluate these publications to determine whether they are relevant and should be cited. There is no requirement to cite these works unless the editor has indicated otherwise.

[Note: HTML markup is below. Please do not edit.]

Reviewers' comments:

Reviewer’s Responses to Questions

**Comments to the Author**

1. Is the manuscript technically sound, and do the data support the conclusions?

The manuscript must describe a technically sound piece of scientific research with data that supports the conclusions. Experiments must have been conducted rigorously, with appropriate controls, replication, and sample sizes. The conclusions must be drawn appropriately based on the data presented.

Reviewer #1: Yes

Reviewer #2: Partly

Reviewer #3: No

**********

2. Has the statistical analysis been performed appropriately and rigorously?

Reviewer #1: Yes

Reviewer #2: I Don't Know

Reviewer #3: No

**********

3. Have the authors made all data underlying the findings in their manuscript fully available?

The PLOS Data policy requires authors to make all data underlying the findings described in their manuscript fully available without restriction, with rare exception (please refer to the Data Availability Statement in the manuscript PDF file). The data should be provided as part of the manuscript or its supporting information, or deposited to a public repository. For example, in addition to summary statistics, the data points behind means, medians and variance measures should be available. If there are restrictions on publicly sharing data—e.g. participant privacy or use of data from a third party—those must be specified.

Reviewer #1: Yes

Reviewer #2: Yes

Reviewer #3: No

**********

4. Is the manuscript presented in an intelligible fashion and written in standard English?

PLOS ONE does not copyedit accepted manuscripts, so the language in submitted articles must be clear, correct, and unambiguous. Any typographical or grammatical errors should be corrected at revision, so please note any specific errors here.

Reviewer #1: Yes

Reviewer #2: No

Reviewer #3: No

**********

5. Review Comments to the Author

Please use the space provided to explain your answers to the questions above. You may also include additional comments for the author, including concerns about dual publication, research ethics, or publication ethics. (Please upload your review as an attachment if it exceeds 20,000 characters)

Reviewer #1: Compulsory revisions

Abstract

The graphical abstract needs proper labelling so that readers can quickly understand the process, key components, or findings.

Introduction

• The introduction part contains too much technical detail, and dense information such as laser energy in nJ, types of filters without context. It is better to write in a clear form for better interation with the reader.

• The WHO death statistics quoted is old, use a recent report.

• There is inconsistent flow in the write up: the transition from classical methods to deep learning is abrupt. Then the Eidos system is introduced without first motivating the gap that it fills. Give a brief introduction of Eidos and ASC.

Methods

• Figure 1 and 2 looks blurred, replace with clearer images

Results and Discussion

• You mentioned that using the INF3 model, digitalized blood smear image data were recognized with a high degree of similarity.

Give a clear explanation of this model before reporting it in this section.

• You mention using SWOT analysis, can you give the full meaning and a brief description of the analysis.

• Figure 13 needs to be properly labelled at the X and Y axis.

Reviewer #2: Dear Editor,

Please find below, my comments and suggestions on the reviewed manuscript:

General:

The manuscript describes a study which employed an automated system-cognitive analysis of digitized blood smears as a diagnostic tool for malaria. This is a step forward in malaria diagnosis. However, there is the need for a critical revision of the manuscript to attend to the reporting style in order to ensure the validity and reliability of the findings. There is also a need for clarity and consistency throughout the manuscript so as to strengthen its overall quality and contribution to the understanding of the use of automation cognitive system for digital blood smears in malaria diagnostics.

• The English language quality of the manuscript needs improvement to make for better reading.

• The technical language of the manuscript needs to be revised to make it more scientifically sound, and easily comprehensible across the readership community.

• The authors need to state/include at least, the genus of the causative agent(s) of malaria (i.e., Plasmodium) in the manuscript. This can be used interchangeably with malaria where applicable.

Graphical Abstract:

• Line 1: The authors should consider including the highlighted words: “…. by species of Plasmodium parasites …”

• Line 2: The genus name, Anopheles, should be italicized; and subsequently, throughout the manuscript.

Introduction:

• Line 1: “contract” is a better word for ‘’obtain’’. Also consider including the highlighted texts: “…. female Anopheles mosquitoes carrying …..”

• Lines 3-5: The statement “According to the ……., 438,000 … in 2015, … 620,000 … in 2017, …. 300-500 million …… [2]” is obsolete and should be replaced with more recent data. The authors are advised to refer to: (263 million and 597,000 malaria cases and deaths respectively, in 2023 [World Malaria Report, 2024]).

• Line 12: The authors need to be more specific with the statement “To count the number of infected cells in a single blood film ….” by indicating the type of cells and blood film (thin or thick)?

• Lines 16-19: The statement “Rapid tests …….. impact patient treatment [7].” should be recast for better comprehension. For instance, “Rapid tests” could be re-written as “rapid diagnostic tests (RDTs)”

• Third paragraph: The authors need to be more specific with the statement: ”Sophisticated image processing ….. automate malaria detection [8]“. ….. using shape, color, intensity, size and texture …..“. The question is: Of what? The malaria parasite, or blood cells?

• Third paragraph: The authors need to be more specific with the statement: ”Lasers …. the cells of interest from blood smears [8]“. The authors should state the cells of interest.

• Third paragraph: Consider revising the statement as highlighted: ”Images of diseased (infected) and uninfected red blood cells (RBCs) … [16, 17]“.

• Third paragraph: Consider revising the statement as highlighted: ” … films that can identify low parasitemia [21, 22]“.

• Third paragraph. If what the authors mean by tiny in the phrase? … with identifying tiny malaria parasites in blood … [24]“ is in regard to the malaria parasite density, then they should consider using low.

• Page 4: Line 2: “….. and solving several other problems.” should be referenced.

• Last paragraph. Consider the following “The present work aims to ….”.

Methods

2.1. Formalization of the subject Area:

AURO University is in India. However, it is not in Mumbai, Maharashtra, as stated by the authors, but rather in Surat, Gujarat.

• Consider revising “…. 550 images of infected and uninfected blood smears ….” as: “…. 550 images of malaria (Plasmodium)-positive and negative blood smears, ….”

• ….. can be used for training and testing. Respectively???

• “Examples of malaria-unaffected ….” to “Examples of malaria-negative ….”

• “… examples of affected …. are demonstrated ….” to “… examples of malaria-positive …. are shown ….”

• Consider revising Titles of Figs. 1 and 2 as Malaria/Plasmodium-negative blood smear samples and Malaria/Plasmodium-positive blood smear samples, respectively.

• Page 5, lines 7/8: “The spectra ….. are then compared with those of classes”. What classes? According to the Eidos system or what?

• Last paragraph: “…. starting with column Dare …” to “…. starting with column D are …”

2.2. Synthesis of statistical and system-cognitive models:

• Consider revising Title of Fig. 6 as Matrix of conditional percentage distributions.

• Page 8: Line 9: What is the full meaning of the acronym INF3?

• Page 9: Line 5: Consider replacing ‘sick’ with ‘infected’ and ‘healthy’ with ‘uninfected’.

Results and Discussion

3.1. Results of recognition of the examined blood smears:

• Fig. 9: Given that a malaria recognition accuracy rate of 100% was recorded with several of the studied smears, the authors should consider showing at least, one of such in Fig. 9.

• “All 100% of infected ……” Better to use either of the two (i.e., All or 100%).

• Consider changing “The circular histogram of malaria ….” to “The pie chart of malaria …...”

• Fig. 10: This is not a circular histogram, but rather, a pie chart! Also, the , (commas) in the listed values should be replaced by . (i.e., dots).

• Page 9, Last paragraph: Consider replacing “The right column ……. colors” with “Legend:”. This should be placed atop the column showing the different colors and values.

• Fig. 11: The data shown here is a duplication of Fig. 9! Moreover, Fig. 11 is supposed to show the results of recognition of digitized blood smears of healthy patients!

3.2. Results of the selection of light spectra for the study:

• Consider revising “The Eidos system allows you to determine …..” as “The Eidos system allows for the determination of …” and “The effect of light spectra is …..” as “The result of this is …….”

• Page 11, Line 1: The full meaning of SWOT (i.e., Strengths, weaknesses, Opportunities, and threats) should be stated in parenthesis.

Conclusion

• Line 6: i.e., infected blood masks?

Reviewer #3: The manuscript proposes using the “Eidos” intelligent system to diagnose malaria from digitized thin blood smear images by converting images into “12 light spectra” and classifying them via an automated system-cognitive (ASC) approach. The authors report an “average similarity value” of 66.965% for infected samples, “no false positives” for healthy samples, and a processing time of ~10 seconds for 18 new images.

While rapid, low-cost diagnostic support is an important goal, the current study has substantial methodological, reporting, and reproducibility limitations that prevent a reliable assessment of performance or utility- see attached document please.

**********

6. PLOS authors have the option to publish the peer review history of their article (what does this mean?). If published, this will include your full peer review and any attached files.

If you choose “no”, your identity will remain anonymous but your review may still be made public.

**Do you want your identity to be public for this peer review?**   For information about this choice, including consent withdrawal, please see our Privacy Policy.

Reviewer #1: **Yes:**   Adedolapo Blessing Olorunfemi

Reviewer #2: No

Reviewer #3: No

**********

[NOTE: If reviewer comments were submitted as an attachment file, they will be attached to this email and accessible via the submission site. Please log into your account, locate the manuscript record, and check for the action link "View Attachments". If this link does not appear, there are no attachment files.]

While revising your submission, please upload your figure files to the Preflight Analysis and Conversion Engine (PACE) digital diagnostic tool, https://pacev2.apexcovantage.com/. PACE helps ensure that figures meet PLOS requirements. To use PACE, you must first register as a user. Registration is free. Then, login and navigate to the UPLOAD tab, where you will find detailed instructions on how to use the tool. If you encounter any issues or have any questions when using PACE, please email PLOS at figures@plos.org. Please note that Supporting Information files do not need this step.

Attachment Submitted filename: Reviewers Comments.pdf

Attachment Submitted filename: Reviewers report.docx

# Author comment: Author response to Decision Letter 1

**DOI:** 10.1371/journal.pone.0348280.r002

4 Oct 2025

all responses for respected reviewers has been attached as file in submission and all changes have been included in manuscript file revised

Attachment Submitted filename: Response to Reviewers.docx

# Peer review: Decision Letter 1

**DOI:** 10.1371/journal.pone.0348280.r003

**Authors:** Segun OYEDEJI, José Luiz Vieira

18 Feb 2026

PONE-D-25-34548R1

Intelligent Automation Cognitive System for Malaria Diagnosis Using Digital Blood Smears

PLOS One

Dear Dr. A.Saleh,

Thank you for submitting your manuscript to PLOS ONE. After careful consideration, we feel that it has merit but does not fully meet PLOS ONE’s publication criteria as it currently stands. Therefore, we invite you to submit a revised version of the manuscript that addresses the points raised during the review process.

**ACADEMIC EDITOR:**

Dear Dr. A.Saleh,

Please carefully review the comments provided by Reviewer Olaiya Folorunsho and incorporate the necessary revisions into the manuscript, particularly those concerning validation and reproducibility, as well as the discussion of limitations and practical implementation. This decision is  justified on PLOS ONE’s publication criteria and not, for example, on novelty or perceived impact.

Please submit your revised manuscript by Apr 04 2026 11:59PM. If you will need more time than this to complete your revisions, please reply to this message or contact the journal office at plosone@plos.org. When you're ready to submit your revision, log on to https://www.editorialmanager.com/pone/ and select the ’submissions Needing Revision' folder to locate your manuscript file.

Please include the following items when submitting your revised manuscript:

- A letter that responds to each point raised by the academic editor and reviewer(s). You should upload this letter as a separate file labeled 'Response to Reviewers'.
- A marked-up copy of your manuscript that highlights changes made to the original version. You should upload this as a separate file labeled 'Revised Manuscript with Track Changes'.
- An unmarked version of your revised paper without tracked changes. You should upload this as a separate file labeled 'Manuscript'.

-->If you would like to make changes to your financial disclosure, please include your updated statement in your cover letter. Guidelines for resubmitting your figure files are available below the reviewer comments at the end of this letter.

If applicable, we recommend that you deposit your laboratory protocols in protocols.io to enhance the reproducibility of your results. Protocols.io assigns your protocol its own identifier (DOI) so that it can be cited independently in the future. For instructions see: https://journals.plos.org/plosone/s/submission-guidelines#loc-laboratory-protocols. Additionally, PLOS ONE offers an option for publishing peer-reviewed Lab Protocol articles, which describe protocols hosted on protocols.io. Read more information on sharing protocols at https://plos.org/protocols?utm_medium=editorial-email&utm_source=authorletters&utm_campaign=protocols.

We look forward to receiving your revised manuscript.

Kind regards,

José Luiz Fernandes Vieira

Academic Editor

PLOS One

Journal Requirements:

If the reviewer comments include a recommendation to cite specific previously published works, please review and evaluate these publications to determine whether they are relevant and should be cited. There is no requirement to cite these works unless the editor has indicated otherwise.

Please review your reference list to ensure that it is complete and correct. If you have cited papers that have been retracted, please include the rationale for doing so in the manuscript text, or remove these references and replace them with relevant current references. Any changes to the reference list should be mentioned in the rebuttal letter that accompanies your revised manuscript. If you need to cite a retracted article, indicate the article’s retracted status in the References list and also include a citation and full reference for the retraction notice.

[Note: HTML markup is below. Please do not edit.]

Reviewers' comments:

Reviewer’s Responses to Questions

**Comments to the Author**

1. If the authors have adequately addressed your comments raised in a previous round of review and you feel that this manuscript is now acceptable for publication, you may indicate that here to bypass the “Comments to the Author” section, enter your conflict of interest statement in the “Confidential to Editor” section, and submit your "Accept" recommendation.

Reviewer #1: All comments have been addressed

Reviewer #4: (No Response)

**********

2. Is the manuscript technically sound, and do the data support the conclusions?

The manuscript must describe a technically sound piece of scientific research with data that supports the conclusions. Experiments must have been conducted rigorously, with appropriate controls, replication, and sample sizes. The conclusions must be drawn appropriately based on the data presented.

Reviewer #1: Yes

Reviewer #4: (No Response)

**********

3. Has the statistical analysis been performed appropriately and rigorously?

Reviewer #1: Yes

Reviewer #4: (No Response)

**********

4. Have the authors made all data underlying the findings in their manuscript fully available?

The PLOS Data policy requires authors to make all data underlying the findings described in their manuscript fully available without restriction, with rare exception (please refer to the Data Availability Statement in the manuscript PDF file). The data should be provided as part of the manuscript or its supporting information, or deposited to a public repository. For example, in addition to summary statistics, the data points behind means, medians and variance measures should be available. If there are restrictions on publicly sharing data—e.g. participant privacy or use of data from a third party—those must be specified.

Reviewer #1: Yes

Reviewer #4: (No Response)

**********

5. Is the manuscript presented in an intelligible fashion and written in standard English?

PLOS ONE does not copyedit accepted manuscripts, so the language in submitted articles must be clear, correct, and unambiguous. Any typographical or grammatical errors should be corrected at revision, so please note any specific errors here.

Reviewer #1: Yes

Reviewer #4: Yes

**********

6. Review Comments to the Author

Please use the space provided to explain your answers to the questions above. You may also include additional comments for the author, including concerns about dual publication, research ethics, or publication ethics. (Please upload your review as an attachment if it exceeds 20,000 characters)

Reviewer #1: Compulsory revisions

Abstract

The graphical abstract needs proper labelling so that readers can quickly understand the process, key components, or findings.

Comment have been addressed

Introduction

• The introduction part contains too much technical detail, and dense information such as laser energy in nJ, types of filters without context. It is better to write in a clear form for better interation with the reader.

Comment have been addressed

• The WHO death statistics quoted is old, use a recent report.

Comment have been addressed

• There is inconsistent flow in the write up: the transition from classical methods to deep learning is abrupt. Then the Eidos system is introduced without first motivating the gap that it fills. Give a brief introduction of Eidos and ASC.

Comment have been addressed

Methods

• Figure 1 and 2 looks blurred, replace with clearer images

Comment have been addressed

Results and Discussion

• You mentioned that using the INF3 model, digitalized blood smear image data were recognized with a high degree of similarity.

Give a clear explanation of this model before reporting it in this section.

Comment have been addressed

• You mention using SWOT analysis, can you give the full meaning and a brief description of the analysis.

Comment have been addressed

• Figure 13 needs to be properly labelled at the X and Y axis.

Comment have been addressed

Reviewer #4: a) Explicit mention of ethical approvals or data usage permissions for patient samples should be included, even if the dataset is public.

b) Potential clinical implications and how the automated system would support healthcare decision-making should be discussed.

**********

7. PLOS authors have the option to publish the peer review history of their article (what does this mean?). If published, this will include your full peer review and any attached files.

If you choose “no”, your identity will remain anonymous but your review may still be made public.

**Do you want your identity to be public for this peer review?**   For information about this choice, including consent withdrawal, please see our Privacy Policy.

Reviewer #1: **Yes:**   Adedolapo Blessing Olorunfemi

Reviewer #4: **Yes:**   Olaiya Folorunsho

**********

[NOTE: If reviewer comments were submitted as an attachment file, they will be attached to this email and accessible via the submission site. Please log into your account, locate the manuscript record, and check for the action link "View Attachments". If this link does not appear, there are no attachment files.]

To ensure your figures meet our technical requirements, please review our figure guidelines: https://journals.plos.org/plosone/s/figures

You may also use PLOS’s free figure tool, NAAS, to help you prepare publication quality figures: https://journals.plos.org/plosone/s/figures#loc-tools-for-figure-preparation.

NAAS will assess whether your figures meet our technical requirements by comparing each figure against our figure specifications.

Attachment Submitted filename: REVIEWER REPORT.docx

# Author comment: Author response to Decision Letter 2

**DOI:** 10.1371/journal.pone.0348280.r004

10 Apr 2026

Response to Reviewers

Manuscript Title: Intelligent Automation Cognitive System for Malaria Diagnosis Using Digital Blood Smears

Journal: PLOS ONE

Dear Academic Editor and Reviewers,

We sincerely thank you for your careful evaluation of our manuscript and for the constructive comments. We appreciate the opportunity to revise our work. We have carefully considered all comments and revised the manuscript accordingly. All changes have been incorporated into the revised manuscript and highlighted in the tracked-changes version.

Below, we provide a point-by-point response.

Response to the Academic Editor

Fund statement

The funding has been removed, as the King Salman Center for Disability Research does not currently support this research due to exceeding the contractual deadline for completing the publication process.

Comment:

Please carefully review the comments provided by Reviewer Olaiya Folorunsho and incorporate the necessary revisions into the manuscript, particularly those concerning validation and reproducibility, as well as the discussion of limitations and practical implementation.

Response:

Thank you for this guidance. We revised the manuscript to strengthen the presentation of validation, reproducibility, limitations, and practical implementation. Specifically:

1. We clarified the dataset source and data usage conditions, including the public availability of the malaria blood smear images used in the study.

2. We explicitly stated that the study used a publicly available, de-identified dataset and that no institutional review board approval or informed consent was required.

3. We expanded the description of the digitization and analysis workflow, including the role of the Aidos system, the use of 12 spectral ranges, and the practical steps required for importing and processing images.

4. We added a clearer discussion of reproducibility-related issues, including file format requirements, image size constraints, computational limitations, and the need for consistent smear quality and staining.

5. We expanded the discussion of practical implementation by explaining how the system can support preliminary malaria screening and assist healthcare workers, especially in settings with limited access to expert microscopists.

6. We revised the limitations section to better reflect technical and operational constraints.

These revisions were incorporated into the Methods, Discussion, Limitations, Ethics statement, Source Data, and Conclusion sections.

Response to Reviewer 1

We thank Reviewer-1 for the positive assessment and for confirming that the previous comments have been addressed.

General comment

The graphical abstract needs proper labelling so that readers can quickly understand the process, key components, or findings.

Comment have been addressed

• The introduction part contains too much technical detail, and dense information such as laser energy in nJ, types of filters without context. It is better to write in a clear form for better interation with the reader.

Comment have been addressed

• The WHO death statistics quoted is old, use a recent report.

Comment have been addressed

• There is inconsistent flow in the write up: the transition from classical methods to deep learning is abrupt. Then the Eidos system is introduced without first motivating the gap that it fills. Give a brief introduction of Eidos and ASC.

Comment have been addressed

• Figure 1 and 2 looks blurred, replace with clearer images

Comment have been addressed

Results and Discussion

• You mentioned that using the INF3 model, digitalized blood smear image data were recognized with a high degree of similarity.

Give a clear explanation of this model before reporting it in this section.

Comment have been addressed

• You mention using SWOT analysis, can you give the full meaning and a brief description of the analysis.

Comment have been addressed

• Figure 13 needs to be properly labelled at the X and Y axis.

Response:

This comment has been addressed. Figure 14 has been updated with clearly defined axis labels to improve interpretability. Specifically, the X-axis now represents the gradations of descriptive scales arranged in descending order of significance (expressed as a percentage of their total number), while the Y-axis represents the cumulative significance of these gradations (expressed as a percentage).

We are grateful for your careful review and positive evaluation of the revised manuscript.

Response to Reviewer 4

We sincerely thank Reviewer-4 for the valuable comments, which helped us improve the manuscript.

Comment 4a

Comment:

Explicit mention of ethical approvals or data usage permissions for patient samples should be included, even if the dataset is public.

Response:

Thank you for this important comment. We have now explicitly clarified the ethical and data-use status of the dataset in the revised manuscript.

Specifically, we added that:

• The blood smear photographs were obtained from a publicly available Kaggle repository;

• The dataset is de-identified, and no patient names or personally identifiable information are provided;

• Therefore, ethical approval and informed consent were not required for the present study;

• Access to the dataset does not require approval from an ethics committee or any other institutional body.

These clarifications have been added in the methods section and further summarized in the ethics statement and third party material.

Added text in the manuscript:

“The blood smear photographs in the Kaggle repository dataset are publicly available, and the patient names are not provided, so no ethical approval or permission to use the samples for research is required.”

We also added in the Ethics statement section:

“This was not a human population study; therefore, approval by the institutional review board and informed consent were not required.”

Comment 4b

Comment:

Potential clinical implications and how the automated system would support healthcare decision-making should be discussed.

Response:

Thank you. We have expanded the manuscript to better explain the clinical relevance and practical role of the proposed system.

In the revised Discussion and Conclusion, we now clarify that the proposed automated system is intended as a preliminary diagnostic support tool, not as a replacement for clinical judgment. The system can support healthcare decision-making in several ways:

The Aidos intelligent system offers the following advantages over traditional microscopy:

The system automatically detects red blood cells and parasites;

Reduces the need for experienced microscopists;

Eliminates laboratory technician bias;

High diagnostic accuracy of blood smears, approximately 97–98%, is achieved, comparable to that of experienced parasitologists;

Analysis is completed in minutes, whereas manual microscopy can take 20–30 minutes per sample;

The system can automatically screen millions of cells, which is virtually impossible to do manually.

We also clarified that the system provides instant diagnostic support and may be especially valuable in Africa and Southeast Asia, where access to specialized laboratory expertise is often limited.

Added/strengthened text in the manuscript includes statements such as:

• “The automated system-cognitive analysis of digitized blood smears provides instant diagnostic support.”

• “It allows medical workers with limited knowledge in microscopy and artificial intelligence to perform diagnostics.”

• “The Aidos intelligent system transforms malaria diagnosis from an expert manual procedure into an automated digital process.”

• “This is especially important for countries in Africa and Southeast Asia, which are remote from laboratories, and where experienced microscopists are often unavailable in endemic regions.”

We also expanded the limitations section to make clear that this system should be viewed as a supportive tool and that practical implementation depends on image quality, correct digitization, and proper operation of the Aidos interface.

Additional Revisions Made in the Manuscript

In addition to the reviewer-specific comments above, we made several improvements for clarity and completeness:

• Clarified the description of the INF3 model, including its role as an information/knowledge model used for recognition and similarity assessment;

The description of the INF3 model has been refined, emphasizing its role as an information/knowledge-based model for recognition and similarity assessment. According to E. V. Lutsenko, the L1 reliability measure for the INF3 model is 0.974. The model demonstrates high performance, with S-precision equal to 1.000 and S-completeness equal to 0.950. Furthermore, the confidence interval for malaria classification is defined by similarity values ranging from >25 to 100 when comparing digital blood smears with reference classes (parasite-infected and healthy samples). Images with similarity scores below 25 are considered unrecognized and excluded from analysis. Notably, no such cases were observed in this study.

• Clarified the meaning of SWOT analysis as “Strengths, Weaknesses, Opportunities, and Threats” and briefly explained its use in the study;

The meaning of SWOT analysis (Strengths, Weaknesses, Opportunities, and Threats) has been explicitly clarified and contextualized within the study. The SWOT diagram for the Active Outcome class represents a feature contribution analysis specific to this class. It illustrates the most significant feature–class relationships derived from ASC analysis, where positive and negative contributions are encoded by color (red/blue), and the thickness of the connecting lines reflects the strength of influence.

• Updated and refined the limitations section;

The limitations section has been expanded and clarified. Specifically, blood smear images must not exceed a resolution of 800 × 600 pixels. Image digitization is conducted across 12 spectral ranges. The Aidos system can process intermediate data up to 2 GB of memory, which limits the number of images processed simultaneously. Additionally, to ensure high reliability, blood smear samples should be standardized in size and shape (e.g., circular) and prepared using consistent staining protocols and color characteristics.

• Ensured that the Data Availability, Ethics statement, and Source Data sections clearly describe the public dataset and its conditions of use;

The Data Availability, Ethics Statement, and Source Data sections have been revised to clearly describe the public dataset used and the associated conditions of use.

• Improved the language and organization of the manuscript to increase readability.

The manuscript has undergone thorough language editing and structural reorganization to improve clarity, coherence, and overall readability.

Closing Statement

We appreciate the reviewers’ and editor’s thoughtful comments, which have helped us improve the manuscript substantially. We hope that the revised version now meets the journal’s requirements and is suitable for publication in PLOS ONE.

Sincerely,

On behalf of all authors

Dr. A. Saleh

Attachment Submitted filename: Responses to Reviewers.docx

# Editor report: Decision Letter 2

**DOI:** 10.1371/journal.pone.0348280.r005

**Authors:** Segun OYEDEJI, José Luiz Vieira, José Luiz Vieira

14 Apr 2026

Intelligent Automation Cognitive System for Malaria Diagnosis Using Digital Blood Smears

PONE-D-25-34548R2

Dear Dra. Saleh

We’re pleased to inform you that your manuscript has been judged scientifically suitable for publication and will be formally accepted for publication once it meets all outstanding technical requirements.

Within one week, you’ll receive an e-mail detailing the required amendments. When these have been addressed, you’ll receive a formal acceptance letter and your manuscript will be scheduled for publication.

An invoice will be generated when your article is formally accepted. Please note, if your institution has a publishing partnership with PLOS and your article meets the relevant criteria, all or part of your publication costs will be covered. Please make sure your user information is up-to-date by logging into Editorial Manager at Editorial Manager® and clicking the ‘Update My Information' link at the top of the page. For questions related to billing, please contact billing support.

If your institution or institutions have a press office, please notify them about your upcoming paper to help maximize its impact. If they’ll be preparing press materials, please inform our press team as soon as possible -- no later than 48 hours after receiving the formal acceptance. Your manuscript will remain under strict press embargo until 2 pm Eastern Time on the date of publication. For more information, please contact onepress@plos.org.

Kind regards,

José Luiz Fernandes Vieira

Academic Editor

PLOS One

Additional Editor Comments

Following a thorough and rigorous evaluation process, we are pleased to note that all the points raised by the reviewers during the previous rounds of revision have been meticulously addressed in your final manuscript. We appreciate the dedication and effort you and your co-authors have put into refining the work, which has significantly contributed to its clarity and scientific robustness.

# Editor report: Acceptance letter

**DOI:** 10.1371/journal.pone.0348280.r006

**Authors:** Segun OYEDEJI, José Luiz Vieira, José Luiz Vieira

PONE-D-25-34548R2

PLOS One

Dear Dr. A.Saleh,

I'm pleased to inform you that your manuscript has been deemed suitable for publication in PLOS One. Congratulations! Your manuscript is now being handed over to our production team.

At this stage, our production department will prepare your paper for publication. This includes ensuring the following:

* All references, tables, and figures are properly cited

* All relevant supporting information is included in the manuscript submission,

* There are no issues that prevent the paper from being properly typeset

You will receive further instructions from the production team, including instructions on how to review your proof when it is ready. Please keep in mind that we are working through a large volume of accepted articles, so please give us a few days to review your paper and let you know the next and final steps.

Lastly, if your institution or institutions have a press office, please let them know about your upcoming paper now to help maximize its impact. If they'll be preparing press materials, please inform our press team within the next 48 hours. Your manuscript will remain under strict press embargo until 2 pm Eastern Time on the date of publication. For more information, please contact onepress@plos.org.

You will receive an invoice from PLOS for your publication fee after your manuscript has reached the completed accept phase. If you receive an email requesting payment before acceptance or for any other service, this may be a phishing scheme. Learn how to identify phishing emails and protect your accounts at https://explore.plos.org/phishing.

If we can help with anything else, please email us at customercare@plos.org.

Thank you for submitting your work to PLOS ONE and supporting open access.

Kind regards,

PLOS ONE Editorial Office Staff

on behalf of

Dr. José Luiz Fernandes Vieira

Academic Editor

PLOS One
